# Sulfonamide-Linked Ciprofloxacin, Sulfadiazine and Amantadine Derivatives as a Novel Class of Inhibitors of Jack Bean Urease; Synthesis, Kinetic Mechanism and Molecular Docking

**DOI:** 10.3390/molecules22081352

**Published:** 2017-08-16

**Authors:** Pervaiz Ali Channar, Aamer Saeed, Fernando Albericio, Fayaz Ali Larik, Qamar Abbas, Mubashir Hassan, Hussain Raza, Sung-Yum Seo

**Affiliations:** 1 Department of Chemistry, Quaid-I-Azam University, Islamabad 45320, Pakistan; mrpervaiz@gmail.com; 2 Department of Organic Chemistry, University of Barcelona, 08028 Barcelona, Spain; albericio@ukzn.ac.za; 3 CIBER-BBN, Networking Centre on Bioengineering, Biomaterials and Nanomedicine, Barcelona Science Park, University of Barcelona, 08028 Barcelona, Spain; 4 Department of Biological Sciences, College of Natural Sciences, Kongju National University, 56 Gongjudehak-Ro, Gongju, Chungnam 314-701, Korea; qamar.abbas.qau@gmail.com (Q.A.); mubashirhassan_gcul@yahoo.com (M.H.); hussain_solangi@yahoo.com (H.R.); dnalove@kongju.ac.kr (S.-Y.S.); 5 Department of Physiology, University of Sindh, Jamshoro 76080, Pakistan

**Keywords:** jack bean urease inhibition, kinetic mechanism, molecular docking, sulfonamides, drug derivatives, drug discovery

## Abstract

Sulfonamide derivatives serve as an important building blocks in the drug design discovery and development (4D) process. Ciprofloxacin-, sulfadiazine- and amantadine-based sulfonamides were synthesized as potent inhibitors of jack bean urease and free radical scavengers. Molecular diversity was explored and electronic factors were also examined. All 24 synthesized compounds exhibited excellent potential against urease enzyme. Compound **3e** (IC_50_ = 0.081 ± 0.003 µM), **6a** (IC_50_ = 0.0022 ± 0.0002 µM), **9e** (IC_50_ = 0.0250 ± 0.0007 µM) and **12d** (IC_50_ = 0.0266 ± 0.0021 µM) were found to be the lead compounds compared to standard (thiourea, IC_50_ = 17.814 ± 0.096 µM). Molecular docking studies were performed to delineate the binding affinity of the molecules and a kinetic mechanism of enzyme inhibition was propounded. Compounds **3e**, **6a** and **12d** exhibited a mixed type of inhibition, while derivative **9e** revealed a non-competitive mode of inhibition. Compounds **12a**, **12b**, **12d**, **12e** and **12f** showed excellent radical scavenging potency in comparison to the reference drug vitamin C.

## 1. Introduction

Ureases (EC 3.5.1.5; urea amidohydrolase) belong to a super group of amidohydrolases and phosphotriestreases, heteropolymeric enzymes with the dynamic site containing two nickel (II) atoms, found in extensive amounts in nature among plants, microbes, organisms, green growth and spineless creatures [1,2,3]. Urease-delivering microscopic organisms have harmful effects on human health. Thus, in human beings the ureas of *Helicobacter pylori* cause afflictions of the gastrointestinal and urinary tract, for example, stomach disease and peptic ulcers [4,5].

Ciurli et al. proposed a productive and workable enzymatic mechanism [6,7]. The dynamic focus of urease is relied on trapping three water molecules and a hydroxide ion connects between two nickel atoms [8]. Urea possesses two binding sites and is capable of forming hydrogen bonding linkages. The loosely bound urea molecule collapses in a tetrahedral fashion with the release of the carbamate group which eventually cleaves into an ammonia molecule [9]. The release of excess ammonia furnishes the suitable conditions for the survival of *H. pylori* in the stomach [10]. *H. pylori* causes several stomach-related disorders such as urolithiasis, pyelonephritis, hepatic encephalopathy, hepatic unconsciousness and urinary catheter encrustation [11]. The therapeutic treatment of *Helicobacter pylori* has been summarized in a review by Boer et al. [12]. Ureases have a long storied history and research on the toxicity and multifunctionality of ureases is work in progress. Carlini et al. have comprehensively reviewed the mechanism and function of ureases [13]. Urease inhibitors play a pivotal part in the inhibition of the harmful effects of urease enzyme and substantially improve human health [14]. Moreover, urease inhibitors assist in the design of drugs against stomach ulcer disorders [15,16]. Urease has assorted capacities and its inhibition has received exceptional consideration in the course of recent years and numerous urease inhibitors have been described. Among these are hydroxamic corrosive subordinates [17], hydroxyurea [18], hydroxamic acids [19], phosphorodiamidates, imidazoles, for example, rabeprazole, lansoprazole, omeprazole, quinines, thiol derivatives, and phenols, Schiff base and thiourea derivatives [20]. Sulfonamides constitute an important class of organic compounds that possess a broad spectrum of biological activities such as antibacterial, high-ceiling diuretic, hypoglycemic, antithyroid, anti-inflammatory and antiglaucoma effects [21,22,23,24,25,26,27]. Noreen et al. recently reported thiophene-tagged sulfonamides as µg/mL concentration urease inhibitors. Mojzych et al. published pyrazoletriazine- based sulfonamides as dual potent inhibitors of urease and tyrosinase and their synthesized derivatives showed better potential than standard thiourea, with IC_50_ values in the micromolar range [28,29,30].

Sulfonamides can easily be synthesized by the reaction of sulfonyl chlorides with amines in a basic medium. However, a number of different methods for the synthesis of sulfonamides have been described in the literature. The straightforward synthetic routes and extended applications in the pharmaceutical and biological field provide incentive to explore and design the role of commercial drugs based sulfonamides as urease inhibitors.

**Figure 1:**
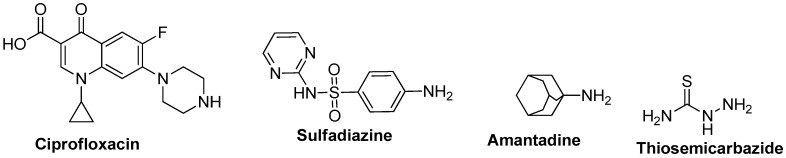
Structures of some synthetic potential urease inhibitor molecules.

Herein, the exploration of novel sulfonamides based drug derivatives, as significant inhibitors of jack bean urease, are described. We thus extended the range of commercial drugs like ciprofloxacin, sulfadiazine, amantadine and thiosemicarbazide (Figure 1).

The potential of commercial drugs as inhibitors of urease has not been explored in enzymology. All three drugs mentioned in Figure 1 are different from one another. However, these drugs contains intriguing structural features which can show strong binding affinity with the target protein. These drugs share a common nucleophilic behavior owing to the presence of electron rich nitrogen atoms. Prior to the current research account, the scope of these drugs has not been extended to urease inhibition. It was hypothesized that variation or structural modification in these commercial drugs could lead to the development of efficient and side effect-free potent inhibitors of urease. In order to test this hypothesis we envisioned uncovering the potential of some marketed drugs. Moreover, the synthetic molecule thiosemicarbazide was also examined to evaluate the role of small organic molecules as inhibitors of urease. Commercial drugs used in this research work are endowed with complex structural groups which could lead to strong binding in the active site of the target protein.

The sulfonamide derivatives have been considered as suitable candidates for the carbonic anhydrase inhibition assay. We took a step further to explore the role as urease inhibitors of sulfonamides, a privileged class of organic compounds.

## 2. Results

**Scheme 1:**
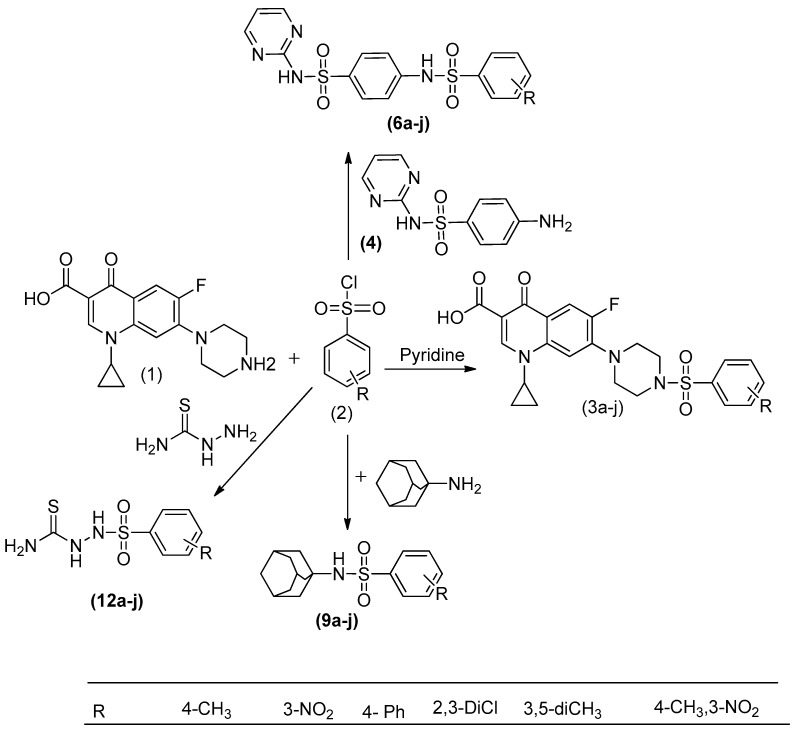
Synthesis of ciprofloxacin-appended sulfonamide derivatives **3a**–**3f**, sulfadiazine drug derivatives **6a**–**6f**, amantadine-linked sulfonamides **9a**–**9f** and thiosemicarbazide sulfonamide derivatives **12a**–**12f**.

The synthetic routes to compounds **3a**–**3f**/**6a**–**6f**/**9a**–**9f**/**12a**–**12f** are shown in Scheme 1. The new series of sulfonamide-based drugs and thiosemicarbazide-based sulfonamides were synthesized in a single step using sulfonyl chloride as an electrophilic reagent. The amine group-bearing versatile drugs ciprofloxacin, amantadine, sulfadiazine and thiosemicarbazide were reacted with suitably substituted sulfonyl chlorides to construct sulfonamide linkages. For sulfadiazine where the amine function is less reactive, reflux conditions in pyridine were required. For the rest, just room temperature. In all cases, the reaction proceeded smoothly with excellent yields (>70%).

### 2.1. Jack Bean Urease Inhibition Assay and Structure Activity Relationship (SAR)

Novel sulfonamide based drug-derivatives were screened in a jack bean urease enzyme inhibition assay. The structural resemblance of the synthesized derivatives with urease enzyme resulted in promising activity. Thiourea was taken as reference drug (Table 1). The synthesized derivatives showed different patterns of chemical reactivity, depending upon the nature of the groups attached with the phenyl ring. The urease inhibition results revealed that these compounds display significant inhibition at low dosis. Among the tested drug derivatives, the ciprofloxacin derivative **3e**, sulfadiazine derivative **6a**, amantadine derivative **9e** and thiosemicarbazide derivative **12d** were found to be the best, with IC_50_ values of 0.0453 ± 0.0016, 0.00223 ± 0.00021, 0.0250 ± 0.00073 and 0.0266 ± 0.0021 µM, respectively. The drug structure played a pivotal part in the urease enzyme inhibition assay. Generally, ciprofloxacin-tagged sulfonamides showed better potency compared to sulfadiazine and amantadine derivatives.

The nature of the group associated with the phenyl ring greatly influenced the biological activity results. Compound **3e** which bears two methyl substituents at the *meta* position is more potent compared to **3a** where they are located at the *para* position. Halogens differ in inhibition results, possibly due to inductive effects, and interestingly a chlorine atom linked at the phenyl ring *ortho* position exhibited better activity. Electron releasing groups showed moderate activity.

### 2.2. Kinetic Mechanism

**Figure 2:**
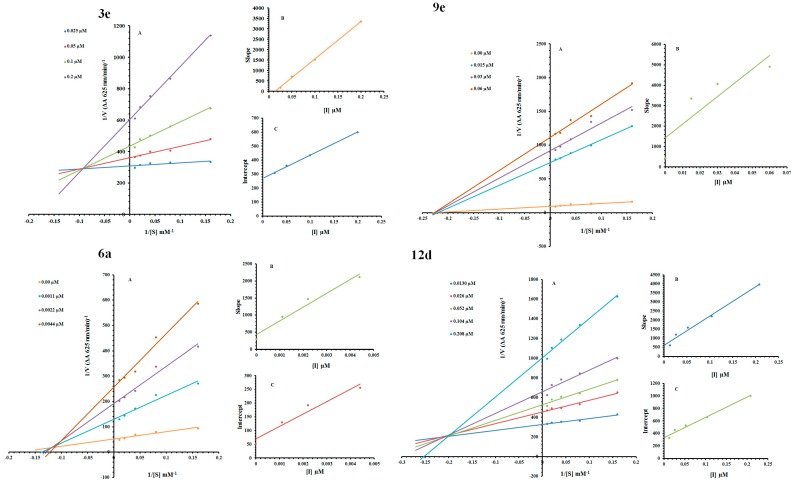
Double reciprocal plot for inhibition of urease in the existence of inhibitor **3e**: (**A**) Doses of **3e** were 0.025, 0.05, 0.01 and 0.02 µM. Substrate urea concentrations were 100, 50, 25, 12.5, 6.25 and 3.125 mM; (**B**) The graph indicates the plot of the slope (**C**) the vertical intercepts versus inhibitor **3e** concentrations to determine inhibition constants. Double reciprocal plot plots for inhibition of urease in the presence of Compound **6a**: (**A**) Concentrations of **6a** were 0.00, 0.0011, 0.0022 and 0.0044 µM. Substrate urea concentrations were 100, 50, 25, 12.5, 6.25, and 3.125 mM; (**B**) The insets represent the plot of the slope (**C**) the vertical intercepts versus inhibitor **6a** concentrations to determine inhibition constants. Lineweaver–Burk plots for inhibition of urease in the presence of Compound **9e**: (**A**) Concentrations of **9e** were 0.00, 0.015, 0.03 and 0.06 µM. Substrate urea concentrations were 100, 50, 25, 12.5, 6.25, and 3.125 mM; (**B**) The insets represent the plot of the slope. Lineweaver–Burk plots for inhibition of urease in the presence of Compound **12d**: (**A**) Concentrations of **12d** were 0.013, 0.026, 0.052, 0.104 and 0.208 µM. Substrate urea concentrations were 100, 50, 25, 12.5, 6.25 and 3.125 mM; (**B**) The insets represent the plot of the slope (**C**) the vertical intercepts versus inhibitor **12d** concentrations to determine inhibition constants.

Presently four compounds **3e**, **6a**, **9e** and **12d** (the most potent from each group) were studied for their mode of inhibition against urease. The capability of these inhibitors to restrain the enzyme-substrate complex and free enzyme was resolved as far as enzyme inhibitor (EI) and enzyme substrate complex (ESI) constants individually. The active investigations of the catalyst by the double reciprocal plot of 1/Velocity versus 1/[Substrate] within the sight of various inhibitors doses gave a progression of straight lines as they appear in Figure 2. The outcomes of inhibitor **9e** provide a sequence of conventional positions, all of which crossed at the identical point on the *x*-axis Figure 2, (A). The analysis showed that V_max_ decreased to another dose while that of Km continues as before accordingly to the increment in the doses of compound **9e**. This conduct showed that compound **9e** represses urease non-competitively to frame enzyme inhibitor (EI) complex. An optional plot of slope against centralization of **9e** indicated a consistent EI separation (Ki) Figure 2, (B). The results of Figure 2, (A) showed that compounds **3e**, **6a** and **12d** intersected within the second quadrant. The analysis showed that V_max_ decreased with increasing K_m_ in the presence of increasing concentrations of compounds **3e**, **6a** and **12d**, respectively. This mode of action of inhibitors **3e**, **6a** and **12d** pointed out that they stops the enzyme by a dual method: first competitively by forming a urease inhibitor complex and second by interfering with the urease-substrate (urea)-compound composite, known as noncompetitive mode. The second graph between inhibitor **3e**, **6a** and **12d** versus slope displayed enzyme inhibitor dissociation constant constants *Ki* Figure 2, (B). Whereas enzyme substrate inhibitor dissociation constants *Ki*′ were presented by another graph plotting doses of inhibitors **3e**, **6a** and **12d** versus intercept Figure 2, (C). A lesser value of *Ki* than *Ki′* indicated the solid attachment of enzyme and inhibitors **3e**, **6a** and **12d** that indicates more competitive than noncompetitive behavior (Table 2). The kinetic constant and inhibition constant data are listed in Table 2.

## 3. Discussion

Drug discovery is an intriguing process, which cannot be attempted/faced using just a single strategy. Several groups and important pharmaceutical industries have focused their efforts in the “repurposing” strategy which is basically based on discovering new uses for known drugs [31]. In our current medicinal chemistry programs, we have gone a step further. Thus, we propose the manipulation of known drugs. As proof of concept, we have chosen the sulfonamidation reaction, because sulfonamides are present in many known drugs [32]. As drugs, we have chosen three which contain free amino functions—ciprofloxacin, sulfadiazine and amantadine—and thiosemicarbazide, which although simple, is a motif also present in several drugs [33,34].

### 3.1. Structural Evaluation of the Target Protein

Jack bean urease consists of four domains (A, B, C and D) having different amino acid lengths. Two nickel atoms are also present in the active binding region of the targeted protein. The overall Volume Area Dihedral Angle Reporter (VADAR) analysis showed that the urease architecture contains with 27% helices, 31% β sheets and 41% coils. The predicted Ramachandran plots indicated that 97.5% of residues were present in favored regions which shows the precision and accuracy of phi (φ) and psi (ψ) angles among the coordinates of jack bean urease. The Ramachandran and hydrophobicity graphs are presented in the Supplementary Data (Figures S1 and S2).

### 3.2. Biochemical Properties and Rule of Five (RO5) Validation of Synthesized Compounds

The basic biochemical properties of all compounds **3a**–**f**, **6a**–**f**, **9a**–**f** and **12a**–**f** were predicted using computational tools. Table 3 showed the predicted properties like molecular weight (g/mol), molar refractivity (cm^3^), density (g/cm^3^), polarizability (cm^3^) and polar surface area (PSA) (Å^2^) values of all ligands. The molar refractivity and molecular lipophilicity properties of drug molecules are significant in receptor binding, bioavailability and cellular uptake. It has been shown that PSA is very helpful parameter for drug absorption prediction in drug discovery [35]. The literature study reported the standard values for molar refractivity and molecular weight (40 to 130 cm^3^) and (160 to 480 g/mol), respectively [36,37]. The comparative analyses revealed that the predicted values of all the synthesized compounds were comparable with standard values.

Moreover, irrespective of their higher molecular weight (g/mol) and molar refractivity (cm^3^) values, they showed very good drug-likeness scores. Any single non-ring bond, bonded to a nonterminal heavy (i.e., non-hydrogen) atom is called rotatable bond. The calculated number of rotatable bonds showed molecular flexibility. It has been observed that rotatable bond number is very good descriptor for oral bioavailability drugs [37].

Furthermore, all the compounds were validated by Lipinski’s rules. It has been observed that orally active drugs typically contain no more than 10 hydrogen bond acceptors (HBA) and five hydrogen bond donors (HBD). Moreover, the logP and molecular mass value should be less than 5 and 500 g/mol, respectively. It has been shown that the compounds with HBA and HBD results exceeding these values display poor permeation [38]. Hydrogen-bonding capacity is considered a significant parameter for drug permeability. Our results indicated that the all synthesized compounds possess <10 HBA, <5 HBD and <5 logp values which were comparable with standard values. However, only few compounds possess a molecular weight value >500 g/mol which does not follow the RO5. The reported study showed that molecules with poor absorption are more likely to be observed upon Lipinski violation. However, multiple examples of RO5 violations are known amongst existing drugs [39,40].

### 3.3. Binding Energy Evaluation of Synthesized Compounds

**Figure 3:**
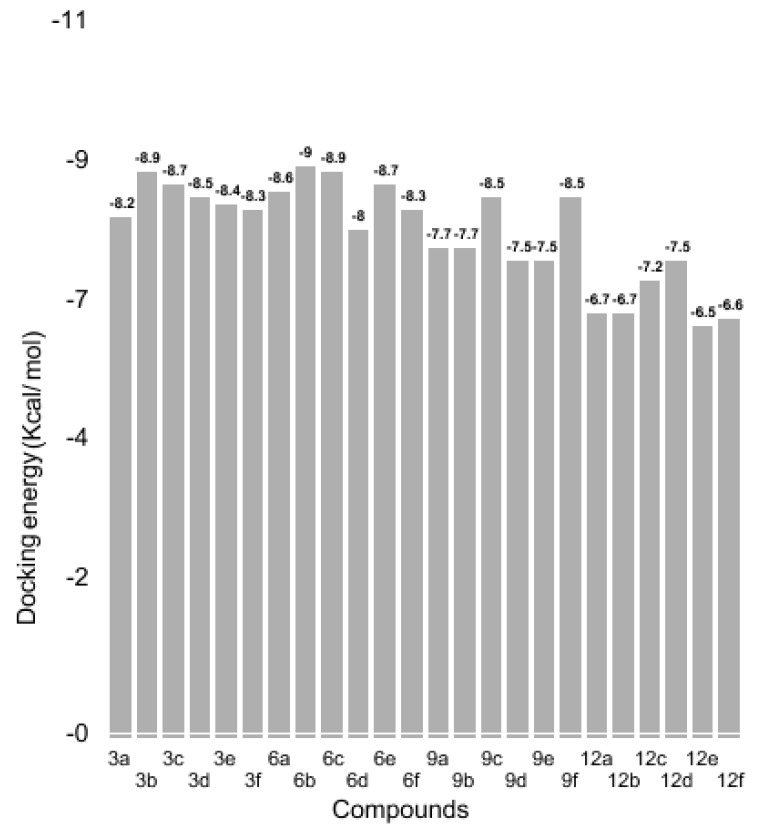
The graphical depiction of docking energy values.

To predict the best fitted conformational position of the synthesized compounds **3a**–**f**, **6a**–**f**, **6a**–**f** and **12a**–**f** against the targeted protein, their generated docked complexes were analyzed on the basis of minimum energy values (Kcal/mol) and bonding interaction patterns (hydrogen/hydrophobic). Among the four different ligand structures, the docking results justified the fact that compounds **3a**–**f** and **6a**–**f** were the most active with better binding energy values as compared to **9a**–**f** and **12a**–**f**. The comparative analysis showed that **6b** has the highest energy value (9.0 Kcal/mol) complex. Compound **6a** also has a good binding energy affinity (8.6 Kcal/mol). The docking results showed that compounds **6a**–**f** have more binding potential against the target protein. Similarly, some other compounds like **3b**, **9c**, **9f**, **12c** and **12d** also possess good binding energy values (−8.9, −8.5, −8.5, −7.2 and −7.5 Kcal/mol, respectively). However, all the other compounds also exhibited good binding energy values (Kcal/mol) against the receptor molecule. A literature study showed that the standard error mean of docking energy for Autodock is 2.5 kcal/mol. Therefore, among all compounds the energy value difference was no more than 2.5 Kcal/mol. However, the selection of compounds was also confirmed on the basis of in vitro analysis to categorize the compounds and further perform a dynamic simulation study. Our computational analysis also showed a good association with the in-vitro study and a graphical depiction is provided in Figure 3.

### 3.4. Urease Binding Pocket and Ligand Pose Analysis

The docked complexes were further analyzed on the basis of hydrogen and hydrophobic binding interactions. Docking analysis showed that the four best compounds were confined in the active binding region of the receptor molecule with different conformational poses. The four best docked compounds’ (**3e**, **6a**, **9c** and **12d**) poses in the binding region of urease with their interaction bonding patterns are shown in (Figure 4). All the other docking complexes are depicted in the Supplementary Data (Figures S3–S22).

### 3.5. Structure Activity Relationship and Interacting Residues

The hydrogen and hydrophobic interactions are very important in drug protein docking. Prior research data showed that hydrogen bonds with donor-acceptor distances of 2.2–2.5 Å are considered as strong bonds, while, bond distances from 2.5 to 3.2 Å are considered moderate, and bond lengths ranging from 3.2–4.0 Å are considered as weak interactions [40]. In detail, the structure activity relationship (SAR) study showed that in **6a** docking two hydrogen bonds were observed against His593, with bond distances of 2.30, 2.60 Å, respectively [41]. However, another showed that the default parameter for the distance between the hydrogen bond donor and the acceptor was 3.2 Å [42]. On average, a hydrogen bond with a distance >3.5 Å is considered as weak and a hydrogen bond with a distance <3.5 Å is considered strong [43]. A single hydrophobic interaction was also seen with Ala636, showing a bond length of 4.03 Å. The amino group of the benzene ring and oxygen moiety of the sulfur interact directly with His593 by a hydrogen bond interaction. Similarly, the benzyl methyl group forms a hydrophobic interaction within the active binding region of the target protein. In the **3e** docking complex three hydrogen bonds were observed. The oxygen and methoxy functional groups of **3e** form hydrogen bonds with Arg439 and Ala636 with bonding distances of 3.06, 2.92 and 4.40 Å, respectively. Compounds **9e** and **12d** are also involved in hydrogen bonding interactions with target protein residues. The oxygen moiety of **9e** forms double hydrogen bonds with Arg439 and His594 with bonding distances of 2.08 and 2.72 Å. Similarly, in **12d** docking a single hydrogen bond was observed between Arg439 and an oxygen molecule of the ligand with a bonding distance 1.78 Å. It has been observed that Arg439 was most common interacting residues in all docking results. Literature data also verified the importance of these residues in bonding with other urease inhibitors which strengthens our docking results. No ligand interaction was observed with the Ni^2+^ due to the bulky ligand structures. However, the interaction patterns of our docking results were correlated with published results which strengthens the significance of our docking experiments [44,45,46,47,48]. The comparative binding energy and SAR analysis showed the significance of **6a** that may be considered as a potent inhibitor targeting jack bean urease.

### 3.6. Radius of Gyration and Protein Stability

**Figure 5:**
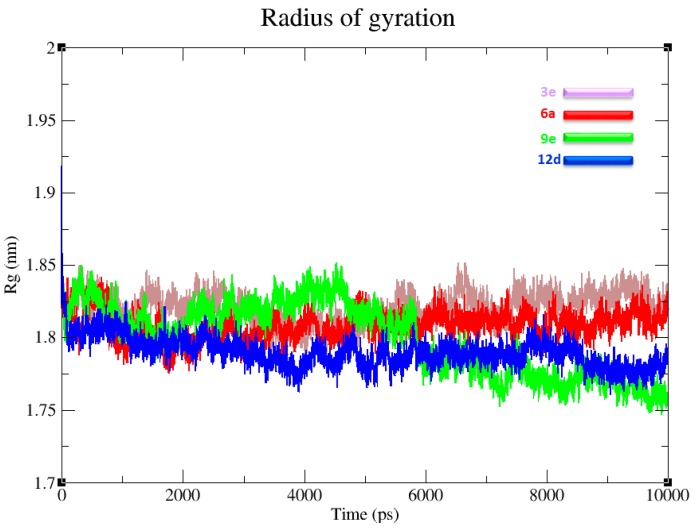
Radius of gyration (Rg) graphs of **3e**, **6a**, **9e** and **12d** docked complexes are indicated in purple, red, green and blue colors, respectively, on a 0–10,000 ps time scale.

The radius of gyration (Rg) prediction was also carefully observed to analyze the compactness of the target protein. The generated graph results indicated that the Rg value is very stable at around 1.83 nm throughout the simulation time (0–10,000 ps). The comparative analysis showed that the **6a** graph line was the most stable and showed little fluctuations compared to other complexes. The Rg time graph showed that the residual backbone and the folding of the receptor protein was steadily stable after binding with the inhibitors (Figure 5). The solvent accessible area (SASA) and χ^2^ distribution/dihedral order of residual fluctuations of all complexes are presented in the Supplementary Data (Figures S23 and S24).

## 4. Materials and Methods

### 4.1. Chemicals and Instruments

All the chemicals and reagents were purchased commercially and used without further purification. All the solvents used were dried and distilled prior to use. Melting points were recorded using a digital Gallenkamp model MPD.BM 3.5 apparatus (SANYO, Tokyo, Japan) and are uncorrected. ^1^H- and ^13^C-NMR spectra were recorded in DMSO-*d*_6_ at 300 MHz and 75 MHz, respectively, using a Bruker (Rheinstetten, Germany) spectrophotometer. IR spectra were recorded on an IR 460 spectrophotometer (Shimadzu, Rheinstetten, Germany) as KBr pellets. Elemental analyses were conducted using a LECO-183 CHNS analyzer (Rheinstetten, Germany). Bioactivities were determined at the Department of Biological Sciences, College of Natural Sciences, Kongju National University. Thin layer chromatography (TLC) was conducted on 0.25 mm silica gel plates (60 F254, Merck, Rheinstetten, Germany). Visualization was made with ultraviolet light. Reagents were obtained commercially and used as received.

### 4.2. Synthetic Procedures

New and modern synthetic protocols were used for the synthesis of sulfonamide derivatives [49]. Sulfonyl chloride (0.5 g, 1.85 mmol) in chloroform (10 mL) was added dropwise to a solution of ciprofloxacin/amantadine/sulfadiazine or thiosemicarbazide (0.28 g, 1.85 mmol not valid for all these substrates) and pyridine (0.77 mL) in chloroform (5 mL) and stirred for 48 h. The reaction mixture was washed successively with 10% citric acid (5–10 mL), 10% NaHCO_3_ (5–10 mL) and water (3–10 mL), dried with MgSO_4_ and concentrated in vacuo. Purification by flash column chromatography (30% hexane–ethyl acetate) yielded the crude compound as a yellow oil. The oil was further purified by recrystallization from chloroform and a few drops of *n*-hexane. The characterization data for the compounds is given below.

### 4.3. Characterization Data

*1-Cyclopropyl-6-fluoro-7-(4-((3-nitrophenyl)sulfonyl)piperazin-1-yl)-4-oxo-1,4-dihydroquinoline-3-carboxylic acid* (**3a**). White solid; yield: 76%; m.p.: 225–227 °C; *R*_f_: 0.63 (1:1 petroleum ether–ethyl acetate); FTIR (neat, cm^−1^): 3135 (C_sp2_-H), 1663 (C=O), 1589, 1541 (Ar C=C), 1487 (N=O), 1251 (C=S); ^1^H-NMR: δ (ppm) 15.09 (s, 1H, COOH), 8.80 (s, 1H, ArH), 8.39 (d, 1H, ArH, *J* = 8.8 Hz), 8.29 (s, 1H, Ar-H), 8.21 (d, 1H, Ar-H, *J* = 8.6 Hz), 7.93 (s, 1H, Ar-H), 7.71–7.67 (m, 1H, ArH), 7.51 (s, 1H, C=CH), 3.69–3.56 (m, 1H, CH), 3.19–3.15 (m, 4H, CH_2_), 2.64–2.59 (m, 4H, CH_2_), 1.11 (d, 4H, CH_2_, *J* = 6.8 Hz); ^13^C-NMR: δ (ppm) 199.0 (ketone C=O), 173.0 (acid C=O), 162.95, 151.1, 149.8, 146.7, 139.6, 134.5, 133.1, 132.6, 130.3, 129.4, 127.0, 114.6, 113.5, 51.7, 42.0, 34.6, 11.7. Anal. Calcd. for C_24_H_24_FN_3_O_5_S: C, 59.39; H, 4.96; N, 8.69; S, 6.62 found: C, 59.12; H, 4.84; N, 8.24; S, 6.48.

*1-Cyclopropyl-6-fluoro-4-oxo-7-(4-tosylpiperazin-1-yl)-1,4-dihydroquinoline-3-carboxylic acid* (**3b**). White solid; yield: 80%; m.p.: 160–162 °C; *R*_f_: 0.61 (1:1 petroleum ether–ethyl acetate); FTIR (neat, cm^−1^): 3030 (C_sp2_-H), 1664 (C=O), 1557, 1527 (Ar C=C), 1258 (C=S); ^1^H-NMR: δ (ppm) 14.09 (s, 1H, COOH), 8.63 (s, 1H, Ar-H), 7.75 (s, 1H, Ar-H), 7.71 (d, 2H, ArH, *J* = 8.3 Hz), 7.56 (s, 1H, C=CH), 7.03 (d, 2H, ArH, *J* = 8.3 Hz), 3.49–3.44 (m, 1H, CH), 3.17–3.11 (m, 4H, CH_2_), 2.14–2.07 (m, 4H, CH_2_), 2.01 (s, 3H, CH_3_), 1.14 (d, 4H, CH_2_, *J* = 6.3 Hz); ^13^C-NMR: δ (ppm) 166.0 (acid C=O), 161.3 (amide C=O), 151.95, 141.8, 138.8, 135.7, 128.3, 124.5, 123.1, 121.6, 120.7, 118.3, 117.0, 115.6, 110.5, 107.0, 52.7, 44.0, 37.6, 9.7. Anal. Calcd. for C_23_H_21_FN_4_O_7_S: C, 53.29; H, 3.96; N, 10.83; S, 6.21 found: C, 53.12; H, 3.84; N, 10.59; S, 6.07.

*1-Cyclopropyl-7-(4-((2,3-dichlorophenyl)sulfonyl)piperazin-1-yl)-6-fluoro-4-oxo-1,4-dihydroquinoline-3-carboxylic acid* (**3c**). White solid; yield: 79%; m.p.: 168–170 °C; *R*_f_: 0.61 (1:1 petroleum ether–ethyl acetate); FTIR (neat, cm^−1^): 3090 (C_sp2_-H), 1667 (C=O), 1561, 1531 (Ar C=C), 1265 (C=S), 735 (C-Cl); ^1^H-NMR: δ (ppm) 14.19 (s, 1H, COOH), 8.80 (s, 1H, ArH), 8.29 (s, 1H, Ar-H), 7.77 (d, 1H, Ar-H, *J* = 8.9 Hz), 7.53 (s, 1H, ArH), 7.34 (d, 1H, Ar-H, 8.9 Hz), 7.28 (s, 1H, C=CH), 3.59–3.55 (m, 1H, *C*H), 3.29–3.26 (m, 4H, CH_2_), 2.54–2.49 (m, 4H, CH_2_), 1.21 (d, 4H, CH_2_, *J* = 6.3 Hz); ^13^C-NMR: δ (ppm) 195.0 (ketone C=O), 171.0 (acid C=O), 169.3162.95, 151.1, 149.8, 146.7, 139.6, 134.5, 133.1, 132.6, 130.3, 129.4, 127.0, 113.6, 111.5, 52.7, 41.0, 31.6, 10.7. Anal. Calcd. for C_29_H_26_FN_3_O_5_S: C, 63.60; H, 4.78; N, 7.68; S, 5.85 found: C, 63.32; H, 4.54; N, 10.59; S, 6.07.

*1-Cyclopropyl-7-(4-((3,5-dimethylphenyl)sulfonyl)piperazin-1-yl)-6-fluoro-4-oxo-1,4-dihydroquinoline-3-carboxylic acid* (**3d**). White solid; yield: 78%; m.p.: 229–231 °C; *R*_f_: 0.65 (1:1 petroleum ether–ethyl acetate); FTIR (neat, cm^-1^): 3150 (C_sp2_-H), 1652 (C=O),, 1593, 1551 (Ar C=C), 1475 (N=O) 1241 (C=S); ^1^H-NMR: δ (ppm): 15.09 (s, 1H, COOH), 12.40 (s, 1H, NH-C=S), 9.09 (d, 2H, Ar-H, *J* = 7.5 Hz), 9.04 (t, 1H, Ar-H, *J* = 7.5 Hz), 8.82 (s, 1H, Ar-H), 7.95 (s, 1H, Ar-H), 7.59 (s, 1H, C=CH), 3.85–3.79 (m, 1H, *C*H), 3.53–3.49 (m, 4H, CH_2_), 3.34–3.30 (m, 4H, CH_2_), 2.30 (s, 6H, CH_3_), 1.32 (d, 4H, CH_2_, *J* = 6.5 Hz); ^13^C-NMR: δ (ppm): 192.0 (ketone C=O), 166.2 (acid C=O), 148.6, 148.3, 144.5, 144.4, 139.5, 137.1, 130.1, 129.3, 122.3, 119.8, 119.7, 111.7, 111.4, 107.4, 46.9, 46.8, 43.1, 36.4, 21.6, 8.1. Anal. Calcd. for C_23_H_20_Cl_2_FN_3_O_5_S: C, 51.14; H, 3.76; N, 7.76; S, 5.91 found: C, 50.92; H, 3.64; N, 7.59; S, 6.07.

*1-Cyclopropyl-6-fluoro-7-(4-((4-methyl-3-nitrophenyl)sulfonyl)piperazin-1-yl)-4-oxo-1,4-dihydroquinoline-3-carboxylic acid* (**3e**). White solid; yield: 80%; m.p.: 160–162 °C; *R*_f_: 0.61 (1:1 petroleum ether–ethyl acetate); FTIR (neat, cm^−1^): 3030 (C_sp2_-H), 1664 (C=O), 1557, 1527 (Ar C=C), 1258 (C=S); ^1^H-NMR: δ (ppm) 14.09 (s, 1H, COOH), 8.63–7.71 (m, 3H, Ar-H), 7.56 (s, 1H, C=CH), 7.03 (d, 2H, ArH, *J* = 8.3 Hz), 3.49–3.44 (m, 1H, CH), 3.17–3.11 (m, 4H, CH_2_), 2.14–2.07 (m, 4H, CH_2_), 2.01 (s, 3H, CH_3_), 1.14 (d, 4H, CH_2_, *J* = 6.3 Hz); ^13^C-NMR: δ (ppm) 190.0 (ketone C=O), 166.0 (acid C=O), 151.95, 138.8, 135.7, 128.3, 124.5, 123.1, 121.6, 120.7, 118.3, 117.0, 115.6, 110.5, 107.0, 52.7, 44.0, 37.6, 9.7. Anal. Calcd. for C_23_H_20_Cl_2_FN_3_O_5_S: C, 51.14; H, 3.76; N, 7.76; S, 5.91 found: C, 50.92; H, 3.64; N, 7.59; S, 6.07.

*7-(4-([1,1′-Biphenyl]-4-ylsulfonyl)piperazin-1-yl)-1-cyclopropyl-6-fluoro-4-oxo-1,4-dihydroquinoline-3-carboxylic acid* (**3f**). White solid; yield: 80%; m.p.: 160–163 °C; *R*_f_: 0.63 (1:1 petroleum ether–ethyl acetate); FTIR (pure, cm^−1^): 3062 (C_sp2_-H), 1667 (C=O), 1558, 1531 (Ar C=C), 1248 (C=S), ^1^H-NMR: δ (ppm) 14.29 (s, 1H, COOH), 8.75 (s, 1H, Ar-H), 7.91 (s, 1H, Ar-H), 7.83 (d, 2H, ArH, *J* = 8.5 Hz), 7.51 (s, 1H, C=CH), 7.43 (d, 2H, ArH, *J* = 8.5 Hz), 7.40–6.92 (m, 5H, ArH), 3.65–3.61 (m, 1H, *C*H), 3.18–3.11 (m, 4H, CH_2_), 2.74–3.67 (m, 4H, CH_2_), 1.22 (d, 4H, CH_2_, *J* = 6.1 Hz); ^13^C-NMR: δ (ppm) 196.0 (C=O of ketone), 170.0 (C=O acid), 150.8, 149.8, 14677, 139.3, 138.5, 137.1, 135.6, 133.7, 129.3, 135.1, 133.6, 131.7, 129.3, 128.0, 117.6, 111.5, 53.7, 45.0, 39.6, 11.7. Anal. Calcd. for C_24_H_23_FN_4_O_7_S: C, 54.34; H, 4.36; N, 10.55; S, 5.99 found: C, 54.12; H, 3.98; N, 10.15; S, 6.07.

*4-Methyl-N-(4-(N-(pyrimidin-2-yl)sulfamoyl)phenyl)benzenesulfonamide* (**6a**). White solid; yield: 81%; m.p.: 223–225 °C; *R*_f_: 0.63 (1:1 petroleum ether–ethyl acetate); FTIR (neat, cm^−1^): 3260 (NH) 3133 (C_sp2_-H), 1587, 1540 (Ar C=C), ^1^H-NMR: δ (ppm) 8.08 (d, 2H, *J* =4.8 Hz, pyrimidyl), 7.09 (t, 1H, *J* = 4.8 Hz, pyrimidinyl), 7.38 (d, 2H, *J* = 8.4 Hz, C_6_H_4_-*meta*), 7.86 (d, 2H, *J* = 8.4 Hz, C_6_H_4_-*ortho*), 7.18 (d, 2H, *J* =8.4 Hz, C_6_H_4_-*meta*), 7.06 (d, 2H, *J* = 8.4 Hz, C_6_H_4_-*ortho*) 9.27 (s, 1H, NH) 2.21 (s, 3H, CH_3_), ^13^C-NMR: δ (ppm) 160.5 (C=N), 158.4, 141.8, 138.8, 135.7, 133.2, 132.5, 130.5, 129.5, 128.3, 120.5, 26.6. Anal. Calcd. For C_17_H_16_N_4_O_4_S_2_: C, 50.47; H, 3.99; N, 13.84; S, 15.85 found: C, 50.12; H, 3.78; N, 13.55; S, 16.67.

*2-Nitro-N-(4-(N-(pyrimidin-2-yl)sulfamoyl)phenyl)benzenesulfonamide* (**6b**). White solid; yield: 80%; m.p.: 160–162 °C; *R*_f_: 0.61 (1:1 petroleum ether–ethyl acetate); FTIR (neat, cm^−1^): 3260 (NH) 3131 (C_sp2_-H), 1586, 1544 (Ar C=C), 1254 (C=S); ^1^H-NMR: δ (ppm) 8.08 (d, 2H, *J* = 4.8 Hz, pyrimidyl), 7.09 (t, 1H, *J* = 4.8 Hz, pyrimidinyl), 7.38 (d, 2H, *J* =8.4 Hz, C_6_H_4_-*meta*), 8.26 (d, 2H, *J* = 8.4 Hz, C_6_H_4_-*ortho*), 7.38 (d, 2H, *J* = 8.4 Hz, C_6_H_4_-*meta*), 7.06 (d, 2H, *J* = 8.4 Hz, C_6_H_4_-*ortho*), 9.30 (s, 1H, NH) ^13^C-NMR: δ (ppm) 181.5 (C=S), 142.8, 139.8, 135.8, 127.3. Anal. Calcd. for C_17_H_16_N_4_O_4_S_2_: C, 44.12; H, 3.02; N, 16.07; S, 14.75 found: C, 43.98; H, 2.99; N, 15.98; S, 14.61.

*N-(4-(N-(pyrimidin-2-yl)sulfamoyl)phenyl)-[1,1′-biphenyl]-4-sulfonamide* (**6c**). White solid; yield: 79%; m.p.: 168–170 °C; *R*_f_: 0.51 (1:1 petroleum ether–ethyl acetate); FTIR (neat, cm^−1^): 3263 (NH) 3132 (C_sp2_-H), 1576, 1542 (Ar C=C), 1253 (C=S); ^1^H-NMR: δ (ppm) 8.18 (d, 2H, *J* = 4.8 Hz, pyrimidyl), 7.19 (t, 1H, *J* = 4.8 Hz, pyrimidinyl), 7.82 (d, 2H, *J* =8.4 Hz, C_6_H_4_-*meta*), 7.76 (d, 2H, *J* = 8.4 Hz, C_6_H_4_-*ortho*), 7.22 (d, 2H, *J* = 8.4 Hz, C_6_H_4_-*meta*), 7.16 (d, 2H, *J* = 8.4 Hz, C_6_H_4_-*ortho*), 7.75–7.39 (m, 5H, Ar), 9.37 (s, 1H, NH) ^13^C-NMR: δ (ppm) 186.5 (C=S), 142.8, 141.7, 139.8, 138.5, 135.8, 133.5, 132.5, 127.3. Anal. Calcd. for C_22_H_18_N_4_O_4_S_2_: C, 56.12; H, 3.89; N, 12.03; S, 13.73 found: C, 55.98; H, 3.76; N, 11.98; S, 13.61.

*2-((2,3-Dichlorophenyl)sulfonyl)hydrazine-1-carbothioamide* (**6d**). White solid; yield: 70%; m.p.: 223–225 °C; *R*_f_: 0.63 (1:1 petroleum ether–ethyl acetate); FTIR (neat, cm^−1^): 3260 (NH) 3133 (C_sp2_-H), 1587, 1540 (Ar C=C), ^1^H-NMR: δ (ppm) 8.08 (d, 2H, *J* = 4.8 Hz, pyrimidyl), 7.09 (t, 1H, *J* = 4.8 Hz, pyrimidinyl), 7.38 (d, 2H, *J* = 8.4 Hz, C_6_H_4_-*meta*), 7.86 (d, 2H, *J* = 8.4 Hz, C_6_H_4_-*ortho*), 7.18 (d, 2H, *J* = 8.4 Hz, C_6_H_4_-*meta*), 7.06 (d, 2H, *J* = 8.4 Hz, C_6_H_4_-*ortho*) 9.27 (s, 1H, NH) 2.21 (s, 3H, CH_3_), ^13^C-NMR: δ (ppm) 179.5 (C=S), 141.8, 138.8, 135.7, 128.3, 26.6. Anal. Calcd. For C_16_H_12_ Cl_2_N_4_O_4_S_2_: C, 41.83; H, 2.64; N, 12.19; S, 13.93 found: C, 41.48; H, 2.46; N, 11.98; S, 13.61.

*2-((3,5-Dimethylphenyl)sulfonyl)hydrazine-1-carbothioamide* (**6e**). White solid; yield: 76%; m.p.: 223–225 °C; *R*_f_: 0.63 (1:1 petroleum ether–ethyl acetate); FTIR (neat, cm^−1^): 3260 (NH) 3133 (C_sp2_-H), 1587, 1540 (Ar C=C), ^1^H-NMR: δ (ppm) 8.08 (d, 2H, *J* = 4.8 Hz, pyrimidyl), 7.09 (t, 1H, *J* = 4.8 Hz, pyrimidinyl), 7.38 (d, 2H, *J* = 8.4 Hz, C_6_H_4_-*meta*), 7.86 (d, 2H, *J* = 8.4 Hz, C_6_H_4_-*ortho*), 7.18 (d, 2H, *J* = 8.4 Hz, C_6_H_4_-*meta*), 7.06 (d, 2H, *J* = 8.4 Hz, C_6_H_4_-*ortho*) 9.27 (s, 1H, NH) 2.21 (s, 3H, CH_3_), ^13^C-NMR: δ (ppm) 179.5 (C=S), 141.8, 138.8, 135.7, 128.3, 26.6. Anal. Calcd. For C_18_H_18_ N_4_O_4_S_2_: C, 51.53; H, 4.54; N, 13.39; S, 15.32 found: C, 51.35; H, 4.36; N, 13.23; S, 15.12.

*2-((4-Methyl-3-nitrophenyl)sulfonyl)hydrazine-1-carbothioamide* (**6f**). White solid; yield: 70%; m.p.: 223–225 °C; *R*_f_: 0.63 (1:1 petroleum ether: ethyl acetate); FTIR (neat, cm^−1^): 3260 (NH) 3133 (C_sp2_-H), 1587, 1540 (Ar C=C), ^1^H-NMR: 8.08 (d, 2H, *J* = 4.8 Hz, pyrimidyl), 7.09 (t, 1H, *J* = 4.8 Hz, pyrimidinyl), 7.38 (d, 2H, *J* = 8.4 Hz, C_6_H_4_-*meta*), 7.86 (d, 2H, *J* = 8.4 Hz, C_6_H_4_-*ortho*), 7.18 (d, 2H, *J* = 8.4 Hz, C_6_H_4_-*meta*), 7.06 (d, 2H, *J* = 8.4 Hz, C_6_H_4_-*ortho*) 9.27 (s, 1H, NH) 2.21 (s, 3H, CH_3_), ^13^C-NMR: δ (ppm) 179.5 (C=S), 141.8, 138.8, 135.7, 128.3, 26.6. Anal. Calcd. for C_18_H_18_ N_4_O_4_S_2_: C, 51.49; H, 4.44; N, 13.29; S, 14.32 found: C, 51.35; H, 4.31; N, 13.21; S, 14.99.

*N-(Adamantan-1-yl)-4-methylbenzenesulfonamide* (**9a**). White solid; yield: 73%; m.p.: 218–222°C; *R*_f_: 0.61 (1:1 petroleum ether–ethyl acetate); FTIR (neat, cm^−1^): 3262 (NH) 3134 (C_sp2_-H), 1587, 1544 (Ar C=C), ^1^H-NMR: δ (ppm) 7.38 (d, 2H, *J* = 8.4 Hz, C_6_H_4_-*meta*), 7.86 (d, 2H, *J* = 8.4 Hz, C_6_H_4_-*ortho*), 9.27 (s, 1H, NH) 2.21 (s, 3H, CH_3_), 1.67–1.79 (m, 6H, CH_2_); 1.85 (s, 6H, CH_2_); 2.29 (s, 3H, CH) ^13^C-NMR: δ (ppm) 141.75, 137.74, 130.49, 129.49, 40.98, 35.80, 29.74, 29.12, 22.4. Anal. Calcd. for C_17_H_23_NO_2_S: C, 66.84; H, 7.58; N, 4.56; S, 10.51 found: C, 66.67; H, 7.41; N, 4.31; S, 10.40.

*N-(Adamantan-1-yl)-4-nitrobenzenesulfonamide* (**9b**). White solid; yield: 84%; m.p.: 164–168 °C; *R*_f_: 0.63 (1:1 petroleum ether–ethyl acetate); FTIR (neat, cm^−1^): 3263 (NH) 3131 (C_sp2_-H), 1584, 1543 (Ar C=C), ^1^H-NMR: δ (ppm) 7.48 (d, 2H, *J* = 8.4 Hz, C_6_H_4_-*meta*), 8.22 (d, 2H, *J* = 8.4 Hz, C_6_H_4_-*ortho*), 9.30 (s, 1H, NH) 2.24 (s, 3H, CH_3_), 1.65–1.77 (m, 6H, CH_2_); 1.84 (s, 6H, CH_2_) ^13^C-NMR: δ (ppm) 143.75, 136.74, 131.49, 128.49, 40.99, 35.85, 29.72, 29.123. Anal. Calcd. for C_16_H_20_N_2_O_4_S: C, 57.11; H, 5.98; N, 8.32; S, 9.52 found: C, 56.99; H, 5.81; N, 7.98; S, 9.41.

*N-(Adamantan-1-yl)-[1,1′-biphenyl]-4-sulfonamide* (**9c**). White solid; yield: 76%; m.p.: 165–171 °C; *R*_f_: 0.55 (1:1 petroleum ether–ethyl acetate); FTIR (neat, cm^−1^): 3263 (NH) 3132 (C_sp2_-H), 1576, 1542 (Ar C=C), ^1^H-NMR: δ (ppm) 7.82 (d, 2H, *J* =8.4 Hz, C_6_H_4_-*meta*), 7.76 (d, 2H, *J* = 8.4 Hz, C_6_H_4_-*ortho*), 7.75–7.39 (m, 5H, Ar), 9.37 (s, 1H, NH), 2.22 (s, 3H, CH_3_), 1.64–1.76 (m, 6H, CH_2_); 1.82 (s, 6H, CH_2_) ^13^C-NMR: δ (ppm) 142.8, 141.7, 139.8, 138.5, 135.8, 133.5, 132.5, 127.3, 41.91, 36.82, 29.71, 28.12. Anal. Calcd. for C_18_H_21_NO_2_S: C, 68.54; H, 6.73; N, 4.45; S, 10.18 found: C, 68.43; H, 5.81; N, 4.28; S, 9.97.

*N-(Adamantan-1-yl)-2,3-dichlorobenzenesulfonamide* (**9d**). White solid; yield: 78%; m.p.: 221–230 °C; *R*_f_: 0.61 (1:1 petroleum ether–ethyl acetate); FTIR (neat, cm^−1^): 3255 (NH) 3135 (C_sp2_-H), 1580, 1542 (Ar C=C), ^1^H-NMR: δ (ppm) 6.64–7.97 (m, 3H, ArH) 9.24 (s, 1H, NH) 2.23 (s, 3H, CH_3_), 1.54–1.66 (m, 6H, CH_2_); 1.72 (s, 6H, CH_2_); ^13^C-NMR: δ (ppm) 142.8, 139.8, 135.8, 133.5, 131.4, 127.3, 42.91, 38.82, 30.71, 27.12. Anal. Calcd. for C_16_H_19_Cl_2_NO_2_S: C, 53.35; H, 5.32; N, 3.87; S, 8.18 found: C, 53.13; H, 5.12; N, 3.78; S, 8.99.

*N-(Adamantan-1-yl)-3,5-dimethylbenzenesulfonamide* (**9e**). White solid; yield: 81%; m.p.: 162–164 °C; *R*_f_: 0.61 (1:1 petroleum ether–ethyl acetate); FTIR (neat, cm^−1^): 3248 (NH) 3156 (C_sp2_-H), 1572, 1532 (Ar C=C), ^1^H-NMR: δ (ppm) 7.61–7.97 (m, 3H, ArH) 9.34 (s, 1H, NH); 2.36 (s, 6H, CH_3_), 2.21 (s, 3H, CH_3_), 1.58–1.68 (m, 6H, CH_2_), 1.73 (s, 6H, CH_2_); ^13^C-NMR: δ (ppm) 141.8, 139.8, 134.8, 127.3, 43.91, 39.82, 31.71, 29.12 27.5. Anal. Calcd. for C_18_H_25_NO_2_S: C, 67.65; H, 7.88; N, 4.38; S, 10.04 found: C, 67.45; H, 7.69; N, 4.09; S, 9.92.

*N-(Adamantan-1-yl)-4-methyl-3-nitrobenzenesulfonamide* (**9f**). White solid; yield: 80%; m.p.: 165–163 °C; *R*_f_: 0.61 (1:1 petroleum ether–ethyl acetate); FTIR (neat, cm^−1^): 3251 (NH) 3152 (C_sp2_-H), 1567, 1537 (Ar C=C), ^1^H-NMR: δ (ppm) 8.10–7.75 (m, 3H, ArH) 9.72 (s, 1H, NH) 2.38 (s, 3H, CH_3_), 2.23 (s, 3H, CH_3_), 1.56–1.64 (m, 6H, CH_2_); 1.72 (s, 6H, CH_2_); ^13^C-NMR: δ (ppm) 144.8, 137.8, 136.8, 131.3, 128.5, 126.5 23.6, 47.91, 43.82, 36.71, 28.12. Anal. Calcd. for C_17_H_22_N_2_O_4_S: C, 58.28; H, 6.34; N, 7.98; S, 9.14 found: C, 58.14; H, 5.97; N, 4.09; S, 8.98.

*2-Tosylhydrazine-1-carbothioamide* (**12a**). White solid; yield (76%; m.p.: 220–222 °C; *R*_f_: 0.63 (1:1 petroleum ether–ethyl acetate); FTIR (neat, cm^−1^): 3264 (NH) 3135 (C_sp2_-H), 1589, 1541 (Ar C=C), 1251 (C=S); ^1^H-NMR: δ (ppm) 7.38 (d, 2H, *J* = 8.4 Hz, C_6_H_4_-*meta*), 7.86 (d, 2H, *J* = 8.4 Hz, C_6_H_4_-*ortho*), 9.27 (s, 1H, NH) 9.31 (s, 1H, NH) 7.1 (s, 2H, -NH_2_); 2.21 (s, 3H, CH_3_); ^13^C-NMR: δ (ppm) 179.5 (C=S), 141.8, 138.8, 135.7, 128.3, 26.6. Anal. Calcd. For C_18_H_11_N_2_O_4_S: C, 45.07; H, 5.21; N, 19.71; S, 15.04 found: C, 44.94; H, 4.97; N, 19.49; S, 14.97.

*2-((4-Nitrophenyl)sulfonyl)hydrazine-1-carbothioamide* (**12b**). White solid; yield: 80%; m.p.: 160–162 °C; *R*_f_: 0.61 (1:1 petroleum ether–ethyl acetate); FTIR (neat, cm^−1^): 3260 (NH) 3131 (C_sp2_-H), 1586, 1544 (Ar C=C), 1254 (C=S); ^1^H-NMR: δ (ppm) 7.38 (d, 2H, *J* = 8.4 Hz, C_6_H_4_-*meta*), 8.26 (d, 2H, *J* = 8.4 Hz, C_6_H_4_-*ortho*), 9.30 (s, 1H, NH) 9.29 (s, 1H, NH) 7.1 (s, 2H, -NH_2_); ^13^C-NMR: δ (ppm) 181.5 (C=S), 142.8, 139.8, 135.8, 127.3. Anal. Calcd. for C_7_H_8_N_4_O_4_S_2_: C, 30.42; H, 2.91; N, 20.29; S, 23.21 found: C, 30.12; H, 2.82; N, 20.13; S, 23.11.

*2-([1,1′-Biphenyl]-4-ylsulfonyl)hydrazine-1-carbothioamide* (**12c**). White solid; yield: 79%; m.p.: 168–170 °C; *R*_f_: 0.51 (1:1 petroleum ether–ethyl acetate); FTIR (neat, cm^−1^): 3263 (NH) 3132 (C_sp2_-H), 1576, 1542 (Ar C=C), 1253 (C=S); ^1^H-NMR: δ (ppm) 7.82 (d, 2H, *J* = 8.4 Hz, C_6_H_4_-*meta*), 7.76 (d, 2H, *J* = 8.4 Hz, C_6_H_4_-*ortho*), 7.75–7.39 (m, 5H, Ar), 9.37 (s, 1H, NH), 9.28 (s, 1H, NH), 7.5 (s, 2H, -NH_2_); ^13^C-NMR: δ (ppm) 186.5 (C=S), 142.8, 141.7, 139.8, 138.5, 135.8, 133.5, 132.5, 127.3. Anal. Calcd. for C_13_H_13_N_3_O_2_S_2_: C, 50.79; H, 4.27; N, 13.65; S, 20.21 found: C, 50.57; H, 4.12; N, 13.19; S, 19.07.

*2-((2,3-Dichlorophenyl)sulfonyl)hydrazine-1-carbothioamide* (**12d**). White solid; yield: 78%; m.p.: 229–231 °C; *R*_f_: 0.65 (1:1 petroleum ether–ethyl acetate); FTIR (neat, cm^−1^): 3258 (NH) 3137 (C_sp2_-H), 1582, 1540 (Ar C=C), 1264 (C=S); ^1^H-NMR: δ (ppm) 6.64–7.97 (m, 3H, ArH) 9.22 (s, 1H, NH), 9.17 (s, 1H, NH), 7.43 (s, 2H, -NH_2_); ^13^C-NMR: δ (ppm) 187.5 (C=S), 142.8, 139.8, 135.8, 133.5, 131.4, 127.3. Anal. Calcd. for C_7_H_7_Cl_2_N_3_O_2_S_2_: C, 28.79; H, 4.27; N, 13.65; S, 20.21 found: C, 50.57; H, 4.12; N, 13.19; S, 19.07.

*2-((3,5-Dimethylphenyl)sulfonyl)hydrazine-1-carbothioamide* (**12e**). White solid; yield: 80%; m.p.: 160–162 °C; *R*_f_: 0.61 (1:1 petroleum ether–ethyl acetate); FTIR (neat, cm^−1^): 3258 (NH) 3156 (C-H), 1578, 1534 (Ar C=C), 1250 (C=S); ^1^H-NMR: δ (ppm) 7.61–7.97 (m, 3H, ArH), 9.34 (s, 1H, NH), 9.28 (s, 1H, NH), 7.76 (s, 2H, -NH_2_), 2.36 (s, 6H, CH_3_); ^13^C-NMR: δ (ppm) 181.5 (C=S), 141.8, 139.8, 134.8, 127.3, 27.5. Anal. Calcd. for C_9_H_13_N_3_O_2_S_2_: C, 41.68; H, 5.06; N, 16.20; S, 24.71 found: C, 41.49; H, 4.90; N, 15.96; S, 24.59.

*2-((4-Methyl-3-nitrophenyl)sulfonyl)hydrazine-1-carbothioamide* (**12f**). White solid; yield: 80%; m.p.: 160–163 °C; *R*_f_: 0.63 (1:1 petroleum ether–ethyl acetate); FTIR (neat, cm^−1^): 3252 (NH) 3150 (C-H), 1568, 1539 (Ar C=C), 1253 (C=S); ^1^H-NMR: δ (ppm) 8.11–7.77 (m, 3H, ArH) 9.74 (s, 1H, NH) 9.68 (s, 1H, NH), 7.73 (s, 2H, -NH_2_), 2.38 (s, 3H, CH_3_); ^13^C-NMR: δ (ppm) 171.5 (C=S), 145.8, 138.8, 137.8, 130.3, 129.5, 127.5 24.6. Anal. Calcd. for C_8_H_10_N_4_O_4_S_2_: C, 33.10; H, 3.47; N, 19.30; S, 22.09 found: C, 32.59; H, 3.32; N, 19.16; S, 23.98.

### 4.4. Urease Inhibition Assay

The jack bean urease action was determined by measuring the ammonia produced by the indophenol method described by Weatherburn. The reaction mixtures, containing 50 µL of buffer (100 mM urea, 10 mM dibasic potassium phosphate, 1 mM EDTA and 10 mM LiCl, pH 8.2), 20 µL of sample and 20 µL of the jack bean urease (5 U/mL) were added in a 96-well plate and the plate was incubated at 37 °C for 30 min. After incubation, 50 μL of phenol reagent (1%, *w*/*v* phenol and 0.005%, *w*/*v* sodium nitroprusside) and 50 μL of alkali reagents (0.5%, *w*/*v* NaOH and 0.1% NaOCl) were added in each well, and again the plate was incubated at 37 °C for 10 min. After the second incubation, the absorbance was checked at 625 nm, using a microplate reader (OPTI_Max_, Tunable, Sunnyvale, CA, USA). All reactions were performed in triplicate. The urease inhibition activity was calculated according to the following formula:

Inhibition (%) = [(*B* − *S*)/*B*] × 100

where *B* and *S* are the optical densities of sample and blank. Thiourea was used as reference.

### 4.5. Kinetic Mechanism Study

Kinetic analysis was carried out to determine the mode of inhibition. Four inhibitors (**3e**, **6a**, **9e** and **12d**) were selected on the basis of the most potent IC_50_ values. The most potent compound was selected from each group. Kinetics were determined by varying the dose of urea in the presence of different doses of compound **3e** (0.025, 0.05, 0.01 and 0.02 µM), compound **6a** (0.00, 0.0011, 0.0022, and 0.0044 µM), compound **9e** (0.00, 0.015, 0.03 and 0.06 µM) and compound **12d** (0.0130, 0.026, 0.052, 0.104 and 0.208 µM). In detail the urea doses were varied between 100, 50, 25, 12.5, 6.25 and 3.125 mM mM for urease kinetics studies and the rest of the procedure was same for all kinetic studies as described in the urease inhibition assay protocol. Maximal initial velocities were determined from the initial linear portion of the absorbances up to 10 min after the addition of enzyme at per minute’s intervals. The inhibition type of the enzyme was assayed by Lineweaver–Burk plots of the inverse of velocities (1/V) versus the inverse of substrate concentration 1/[S] mM^−1^. The EI dissociation constant *Ki* was determined by the secondary plot of 1/V versus inhibitor concentration and the ESI dissociation constant *Ki*′ was calculated from the intercept versus inhibitor concentrations. Urease activity was determined by measuring ammonia production using the indophenol method as reported previously [50]. The results (change in absorbance per min) were recorded at 625 nm using a microplate reader (Optimax Tunable, Sunnyvale, CA, USA).

### 4.6. Free Radical Scavenging Assay

Radical scavenging activity was determined by adjusting the already reported 2,2-diphenyl-1-picrylhydrazyl (DPPH) assay method [51]. The assay arrangement comprised 100 µL of DPPH (150 µM), 20 µL of increasing concentrations of test compounds and the volume was adjusted to 200 µL in each well with DMSO. At a room temperature, the reaction mixture was incubated for 30 min. Ascorbic acid (Vitamin C) was utilized as a model inhibitor. The assay estimations were done by using a micro plate reader (OPTI_Max_, Tunable, Sunnyvale, CA, USA) at 517 nm. The response rates were analyzed and the percent inhibition caused by the presence of the tested inhibitor was calculated. Each concentration was analyzed in three independent experiments run thrice.

### 4.7. Retrieval of Jack Bean Urease Structure from Protein Data Bank (PDB)

The crystal structure of jack bean urease (PDBID 4H9M) was retrieved from the Protein Data Bank (PDB, www.rcsb.org). The selected protein structure was minimized by employing the University of California at San Francisco (UCSF) Chimera 1.10.1 tool [52]. Furthermore, the stereochemical properties of the urease structure and Ramachandran plot and values were generated by Molprobity server [53]. The Discovery Studio 4.1 Client tool was employed to generate the hydrophobicity graph of the target protein [54]. The protein architecture and statistical percentage values of urease helices, β-sheets, coils and turn were predicted using the online server VADAR 1.8 [55].

### 4.8. Molecular Docking of Synthesized Compounds Using AutoDock

Molecular docking experiments were done for all the synthesized ligands **3a**–**f**, **6a**–**f**, **9a**–**f** and **12a**–**f** against urease using different AutoDock 4.2 tools according to the specified instructions [56]. In the receptor (urease) the polar hydrogen atoms and Kollman charges were apportioned and for the ligands, Gasteiger partial charges were designated and non-polar hydrogen atoms were merged. All the torsion angles for all the synthesized ligands were set free to rotate through docking experiments. The docking experiments were performed by considering the receptor as rigid, whereas the ligands were considered as flexible molecules. Grid map values were adjusted at 80 Å × 80 Å × 80 Å on the targeted protein to generate the grid map. The number of docking runs for each docking experiment was set to 100 for the best conformational state. The Lamarckian genetic algorithm (LGA) and empirical free energy function were applied by taking default docking parameters. The docked complexes were further evaluated on lowest binding energy (Kcal/mol) values and hydrogen/hydrophobic bond analysis to choose the best docking pose using Discovery Studio, (Accelrys, San Diego, CA, USA, 4.1) and UCSF Chimera 1.10.1.

### 4.9. Molecular Dynamics Simulations Assay

Based on in vitro analysis, docking energy and conformational pose analysis the four best complexes were selected for molecular dynamic simulations. Groningen Machine for Chemicals Simulations (GROMACS) 4.5.4 package [57] with the GROMOS 53A6 force field [58] were applied on all complexes to interpret the backbone residual flexibility. The receptor and ligands topology files were created by using the GROMOS 53A6 force-field and the online PRODRG Server [59], respectively. Moreover, the receptor–ligand complexes were solvated and placed in the center of a cubic box adjusted to 9 Å distance. Ions were added to neutralize the system charges. Energy minimization (nsteps = 50,000) was done by the steepest descent method (1000 ps). The energy calculation was done by the Particle Mesh Ewald (PME) method [60], whereas covalent bond constraints were calculated by the linear constraint solver (LINCS) algorithm [61]. For final molecular dynamics (MD) setup in a md.mdp file, the time step for integration was adjusted as 0.002 ps, while the cutoff distance for short range neighbor list (rlist) was adjusted to 0.8 nm. The MD run was set to 10,000 ps with nsteps 500,000 for each protein-ligand complexes and trajectories files analysis was done by the Xmgrace tool [47].

## 5. Conclusions

A small library of compounds based on drug derivatives was synthesized using substituted sulphonyl chlorides. All the synthesized compounds were evaluated for jack bean urease inhibition and free radical scavenging activity. Interestingly, all the drug derivatives elicited superior activity against urease enzyme compared to the standard used (thiourea). The kinetic mechanism was determined for the most potent derivatives and it was found that compounds **3e**, **6a**, and **12d** exhibited a mixed type of mode inhibition, while compound **9e** inhibited urease via a non-competitive mechanism. The enzyme inhibition (*Ki*) value was found to be in the micromolar range, which further ascertained the efficacy of the synthesized ligands against the target enzyme. In addition to this, molecular docking studies were performed to explore the non-covalent interactions and chemoinformatics data were also evaluated using the rule of five or Lipinski’s rule and most of the compounds were found to be in good agreement with these rules. Extensive bioinformatics studies were carried out. From the results we conclude that these compounds could serve as lead compounds in designing structural templates for the inhibition of urease enzyme. This is also proof of the concept that simple manipulations of known drugs can led to the discovery of new drugs.

## Figures and Tables

**Figure 4:**
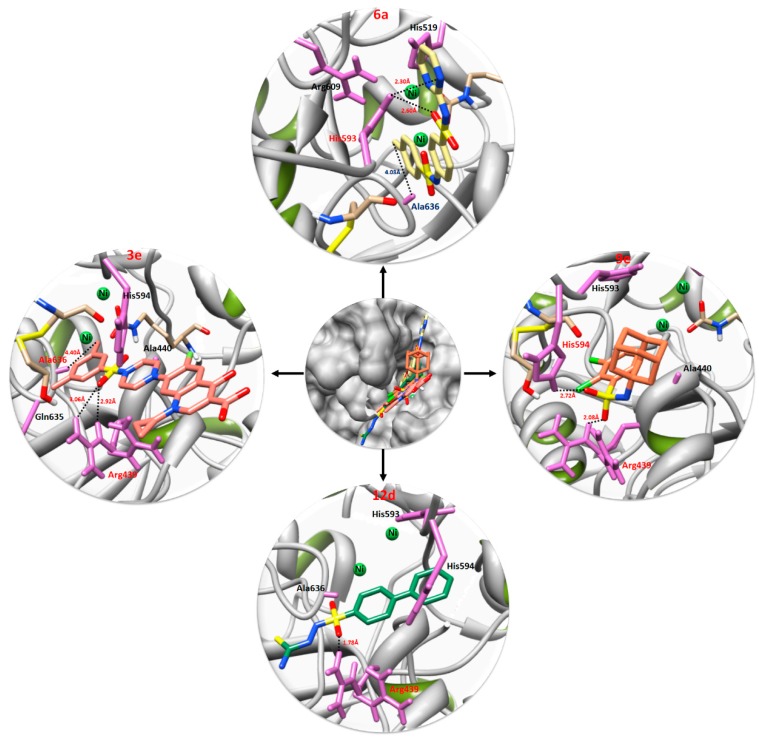
Binding pocket of urease with binding position of the four best synthesized compounds are illustrated in the center. The individual docking results of the best four compounds are labelled in the surrounding circles. In all docking complexes the urease protein is labelled in gray color, with interior color olive drab while the interacting residues are mentioned in purple color. The most active and potent compound **6a** in the docking complex is shown in khaki color, while the other compounds (**3e**, **9e** and **12d**) are represented in salmon, coral and sea green, respectively. The sulfur group in all compounds is highlighted in yellow, while the oxygen and amino groups are represented in red and blue, respectively.

**Table 1:** The inhibitory effect of sulfonamide based drug series on urease (jack bean) activity.

Compound	Urease Activity	Compound	Urease Activity
	IC_50_ ± SEM (µM)		IC_50_ ± SEM (µM)
**3a**	0.081 ± 0.003	**9a**	0.2888 ± 0.015
**3b**	0.078 ± 0.003	**9b**	0.02757 ± 0.001
**3c**	0.110 ± 0.004	**9c**	0.01776 ± 0.000
**3d**	0.082 ± 0.003	**9d**	0.0533 ± 0.001
**3e**	0.045 ± 0.001	**9e**	0.0250 ± 0.000
**3f**	0.073 ± 0.004	**9f**	0.0558 ± 0.001
**6a**	0.002 ± 0.000	**12a**	0.2254 ± 0.006
**6b**	0.233 ± 0.011	**12b**	0.141 ± 0.004
**6c**	0.141 ± 0.007	**12c**	0.186 ± 0.007
**6d**	0.095 ± 0.004	**12d**	0.026 ± 0.002
**6e**	0.174 ± 0.009	**12e**	0.096 ± 0.0040
**6f**	0.127 ± 0.0076	**12f**	0.067 ± 0.0029
Thiourea	17.814 ± 0.096		

SME (standard error of the mean).

**Table 2:** Kinetic parameters of the jack bean urease for urea activity in the presence of different concentrations of **3e**, **6a**, **9e** and **12d**.

Code	Dose (µM)	V_max_ (ΔA/Min)	Km (mM)	Inhibition Type	*Ki* (µM)	*Ki′* (µM)
**3e**	0.025	0.00337	0.606			
	0.05	0.00273	1.923	Mixed-inhibition	0.016	0.195
	0.01	0.00233	3.389			
	0.02	0.001632	5.263			
	0	0.0204	5			
**6a**	0.0011	0.00769	6.666	Mixed-inhibition	0.0011	0.0016
	0.0022	0.00493	6.896			
	0.0044	0.00351	7.518			
	0	0.0114	4.166			
**9e**	0.015	0.00127	4.166	Noncompetitive	0.0225	---
	0.03	0.00108	4.166			
	0.06	0.00085	4.166			
	0.013	0.00299	1.754			
**12d**	0.026	0.00213	2.439	Mixed-inhibition	0.053	0.125
	0.052	0.00194	2.857			
	0.104	0.0016	3.174			
	0.208	0.001	3.773			

V_max_ = the reaction velocity; Km = Michaelis–Menten constant; *Ki* =EI dissociation constant; *Ki′* = ESI dissociation constant; --- = Not calculated.

**Table 3:** Physical properties of the synthesized compounds.

Properties	3a	3b	3c	3d	3e	3f	6a	6b	6c	6d	6e	6f	9a	9b	9c	9d	9e	9f	12a	12b	12c	12d	12e	12f
Mol. weight (g/mol)	485	517	547	539	499	531	404	436	466	466	457	418	305	337	367	359	319	351	245	277	307	298	259	291
No. HBA	6	8	6	6	6	8	6	8	6	6	6	6	3	5	3	3	3	5	4	6	4	4	4	6
No. HBD	1	2	1	1	1	2	2	3	2	2	2	2	1	2	1	1	1	2	4	5	4	4	4	5
Mol. LogP	3.5	2.7	4.9	4.3	3.9	2.9	2.3	1.4	3.7	3.7	3.1	2.7	4.3	3.5	5.7	5.1	4.7	3.8	1.1	0.29	2.5	1.8	1.50	0.5
Mol. PSA (A^2^)	78.3	116	78	78	78	116	102	140	101	101	102	102	41	80	41	41	41	79	73	111	72	73	73	111
Stereo centers	0	0	0	0	0	0	0	0	0	0	0	0	0	0	0	0	0	0	0	0	0	0	0	0
Mol. Vol (A^3^)	470	479	525	482	491	500	327	335	382	382	336	348	312	321	363	321	333	342	202	212	258	211	224	232
Molar Refractivity (cm^3^)	122	124	142	127	127	128	101	102	121	121	106	105	83	85	104	88	88	90	62	64	82	67	67	68
Surface tension (dyne/cm)	69	79	67	73	67	76	74	87	71	71	79	71	51	62	57	58	50	60	65	84	63	73	61	77
Density (g/cm^3^)	1.4	1.6	1.4	1.6	1.4	1.5	1.4	1.6	1.4	1.3	1.6	1.4	1.2	1.4	1.2	1.4	1.2	1.3	1.4	1.6	1.3	1.6	1.3	1.5
Polarizability (cm^3^)	48	49	56	50	50	51	40	40	48	46	42	42	33	33	41	35	35	35	24	25	32	26	26	27
Drug Score	0.92	0.59	1.07	0.80	1.16	0.46	−0.14	−0.64	0.12	0.13	0.04	0.21	−0.85	−0.61	−0.54	−0.82	−0.30	−1.00	−0.06	−0.01	0.12	−0.39	0.23	−0.70
No. Rotatable bonds	5	6	6	5	5	6	6	7	7	7	6	6	3	3	4	3	3	4	4	5	5	4	4	4
Lipinski Rule	Yes	No	No	No	Yes	No	Yes	Yes	Yes	Yes	Yes	Yes	Yes	Yes	Yes	Yes	Yes	Yes	Yes	Yes	Yes	Yes	Yes	Yes

HBA: hydrogen bond acceptors; HBD: hydrogen bond donors; PSA: polar surface area.

## Supplementary Materials

Supplementary Materials are available online.
