# Supplementary material for: Sulfonamide-Linked Ciprofloxacin, Sulfadiazine and Amantadine Derivatives as a Novel Class of Inhibitors of Jack Bean Urease; Synthesis, Kinetic Mechanism and Molecular Docking
Source: Molecules. 2017 Aug 16;22(8):1352. doi: 10.3390/molecules22081352 (PMC6152116; doi:10.3390/molecules22081352)
Supplement: Supplementary file 1 [file molecules-22-01352-s001.pdf]

Supplementary Data

Sulfonamide-linked Ciprofloxacin, Sulfadiazine and Amantadine Derivatives as a Novel Class of Inhibitors of Jack Bean Urease; Synthesis, Kinetic Mechanism and Molecular Docking

Pervaiz Ali Channar [a], Aamer Saeed [*,a], Fernando Albericio [b,c,d], Fayaz Ali Larik [*,a], Qamar Abbas [b], Mubashir Hassan [b], Hussain Raza [b] and Sung-Yum Seo [b]

[a] Department of Chemistry, Quaid-I-Azam University, Islamabad 45320, Pakistan.

[b] Department of Biological Sciences, College of Natural Sciences, Kongju National University, 56 Gongjudehak-Ro, Gongju, Chungnam 314-701, Republic of Korea

[c] Department of Organic Chemistry, University of Barcelona, 08028-Barcelona, Spain

[d] CIBER-BBN, Networking Centre on Bioengineering, Biomaterials and Nanomedicine, Barcelona Science Park, University of Barcelona, Barcelona 08028, Spain

* Email: aamersaeed@yahoo.com, and fayazali@chem.qau.edu.pk Tel +92-51-9064-2128; Fax: +92-51-9064-2241

AutoDock Scoring Function

Combination of knowledge-based and empirical approach:

$$\Delta Gbinding = \Delta Ggauss + \Delta Grepulsion + \Delta Ghbond + \Delta Ghydrophobic + \Delta Gtors$$

where $\Delta Ggauss$: Attractive term for dispersion, two Gaussian functions; $\Delta Grepulsion$: Square of the distance if closer than a threshold value; $\Delta Ghbond$: Ramp function - also used for interactions with metal ions; $\Delta Ghydrophobic$: Ramp function; ΔG tors: Proportional to the number of rotatable bonds

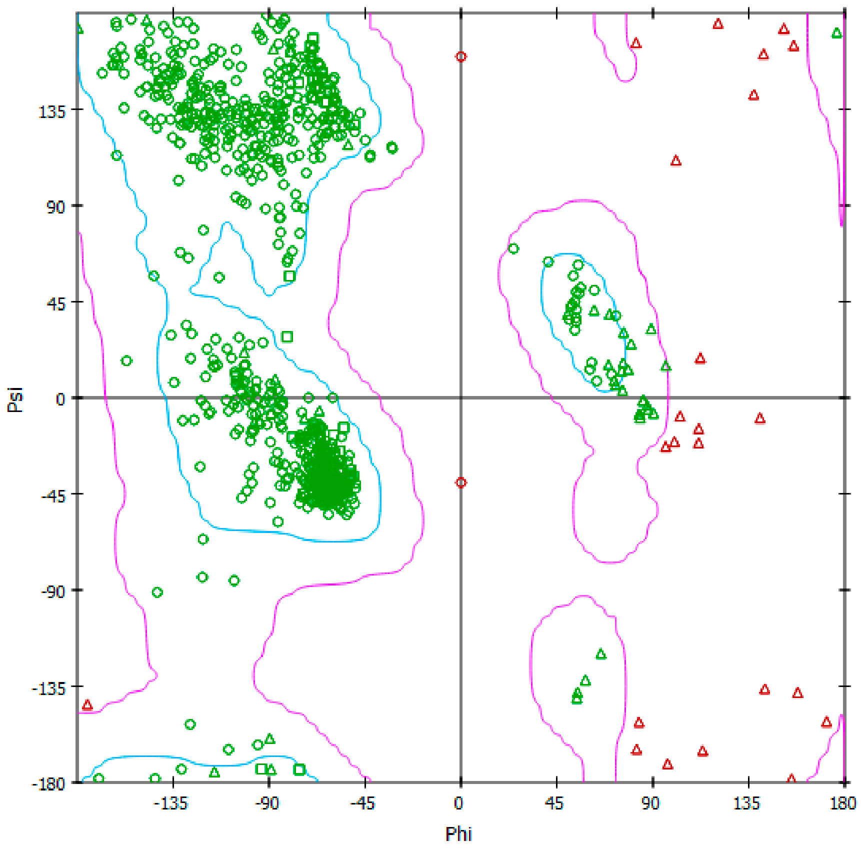

Figure S1. Ramachandran graph of urease

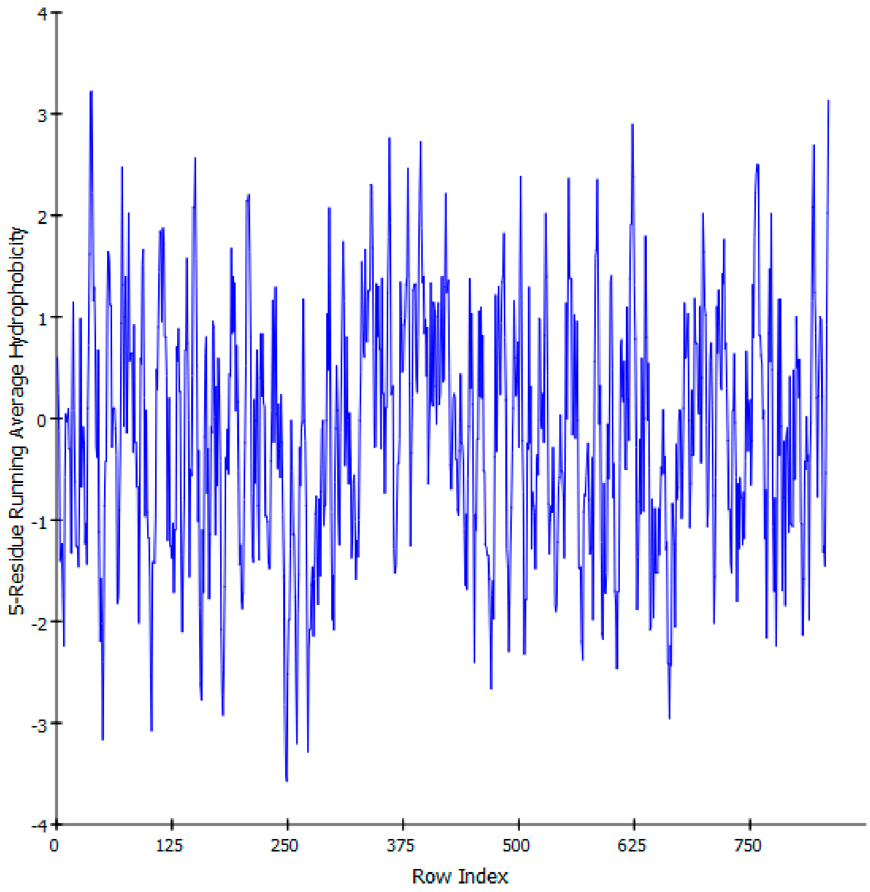

Figure S2. Hydrophobicity graph of urease

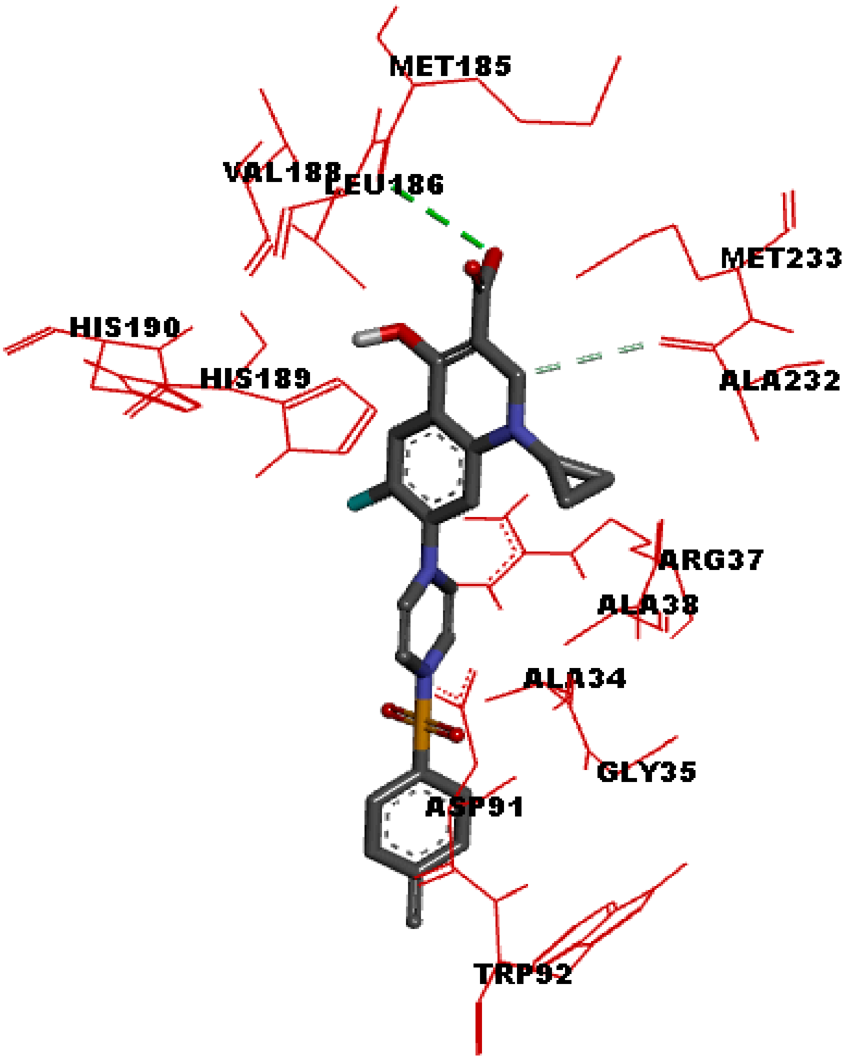

Figure S3. Docking complex **3a**

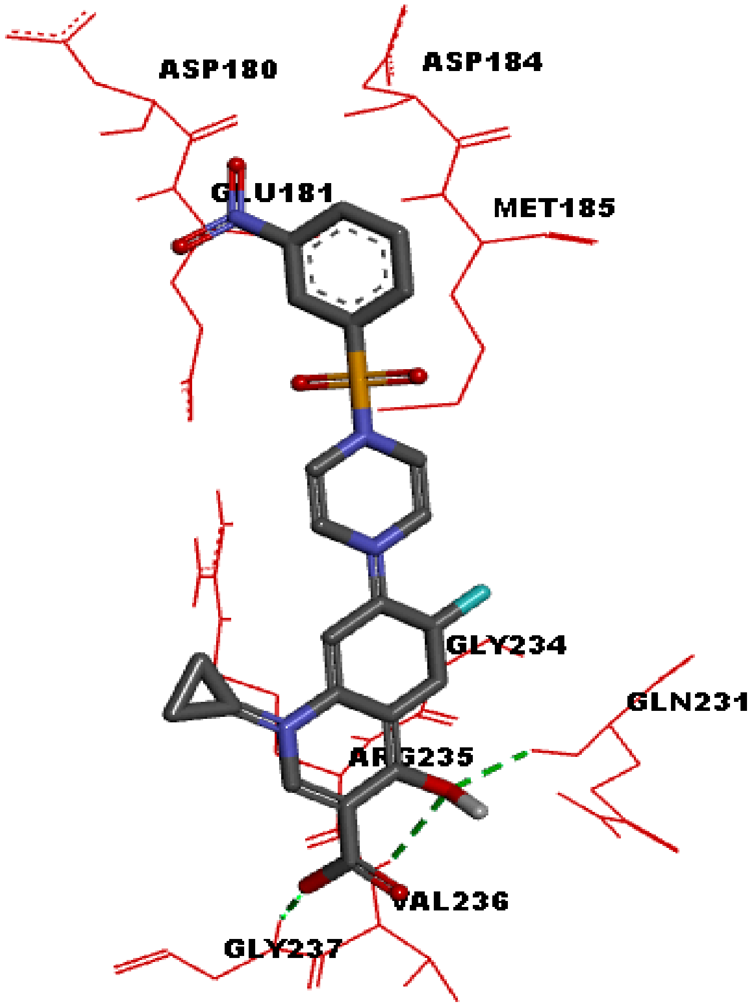

Figure S4. Docking complex **3b**

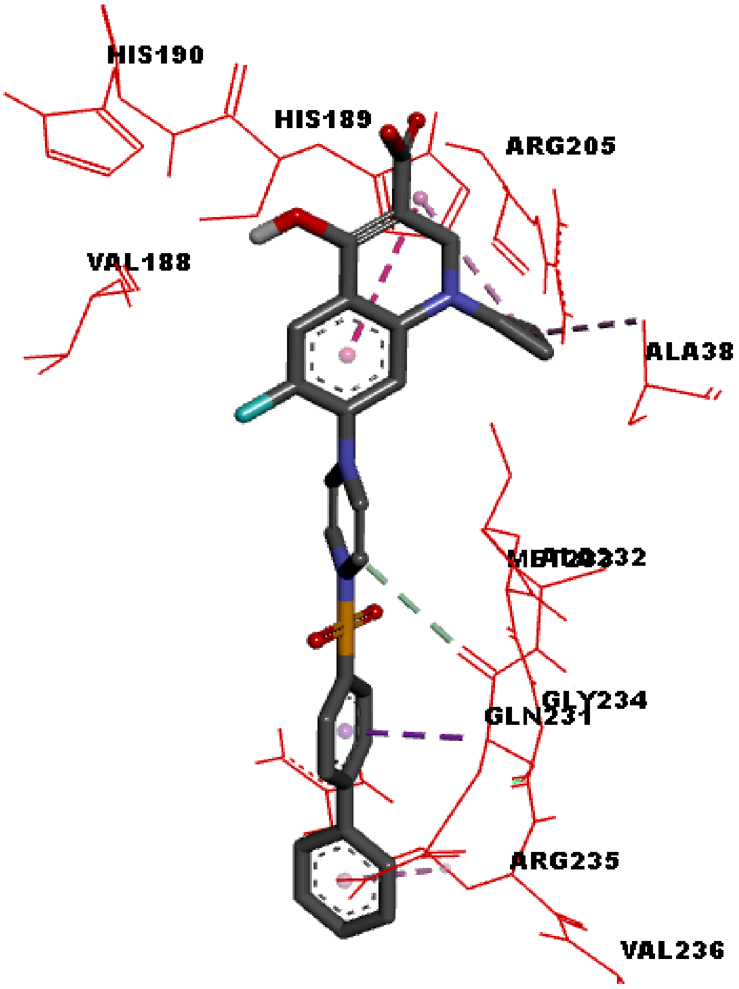

Figure S5. Docking complex **3c**

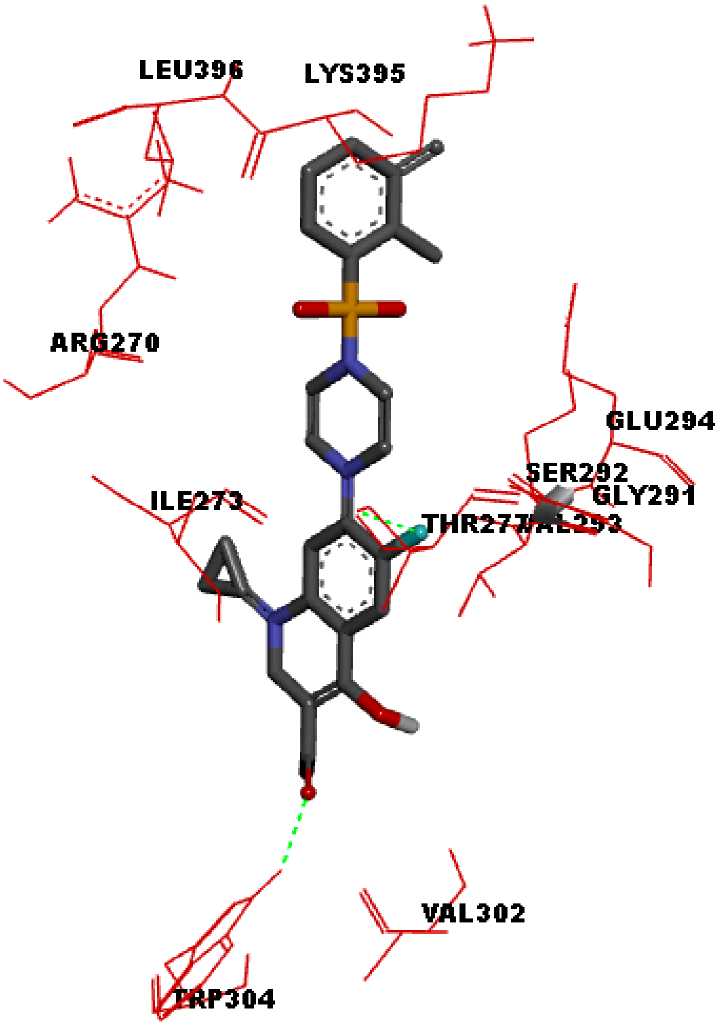

Figure S6. Docking complex **3d**

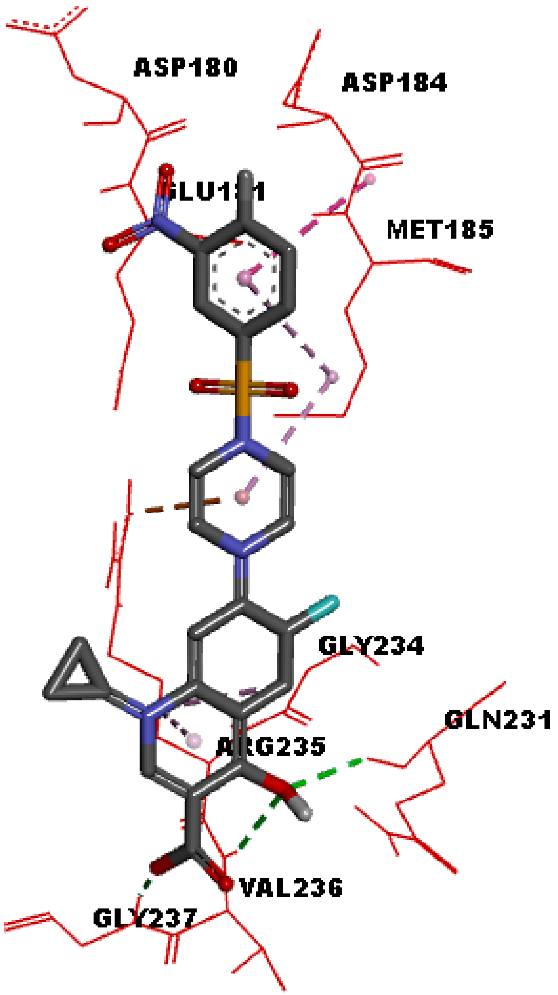

Figure S7. Docking complex **3f**

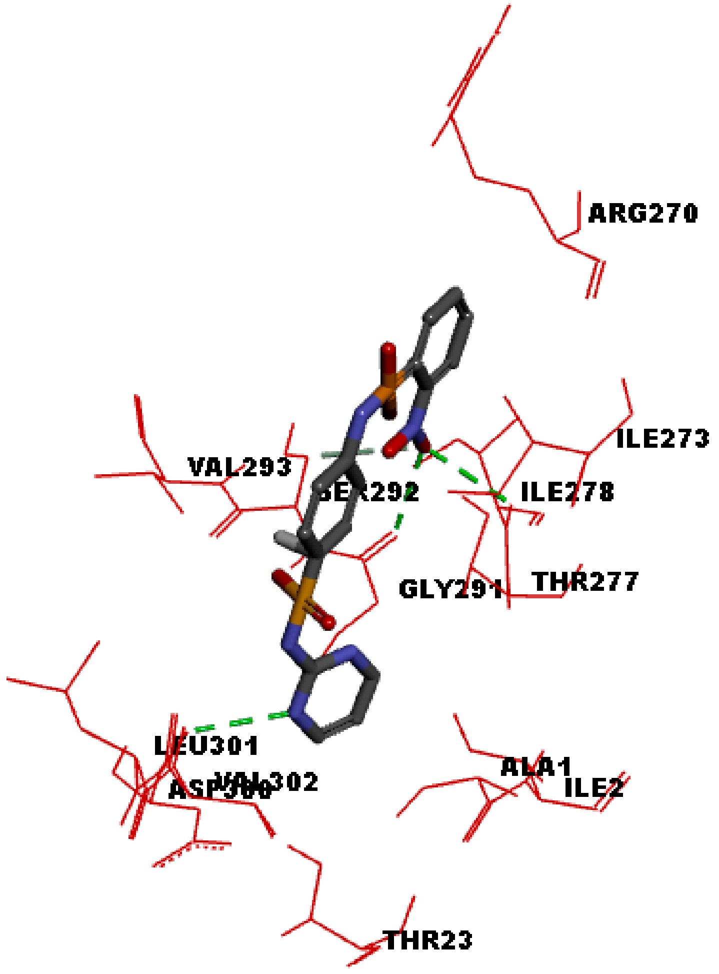

Figure S8. Docking complex **6b**

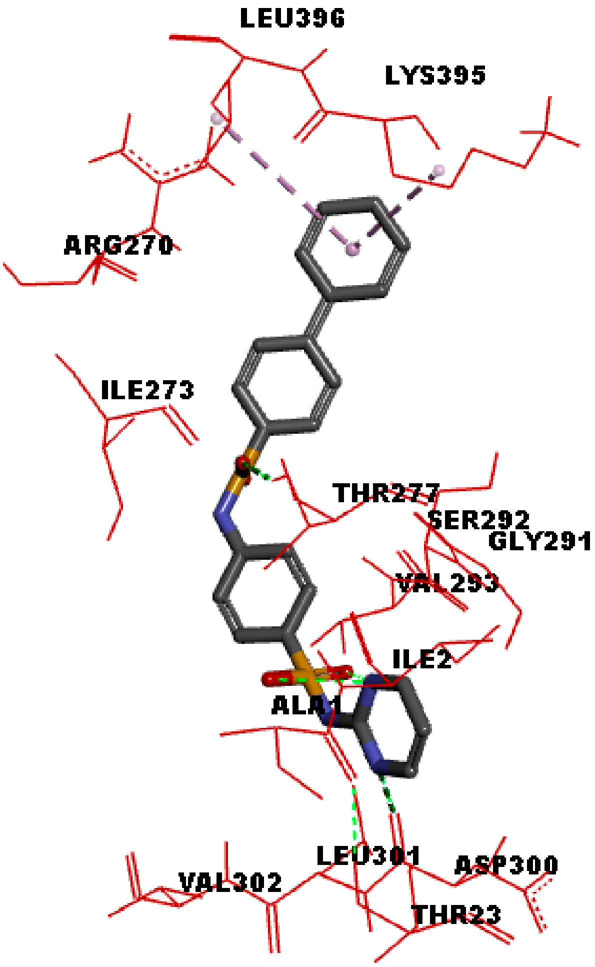

Figure S9. Docking complex **6c**

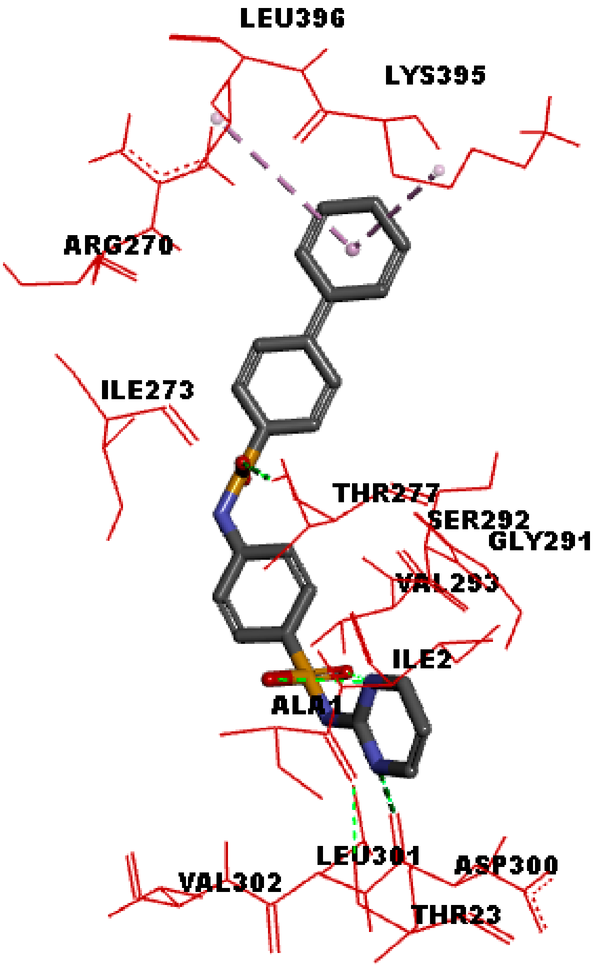

Figure S10. Docking complex **6d**

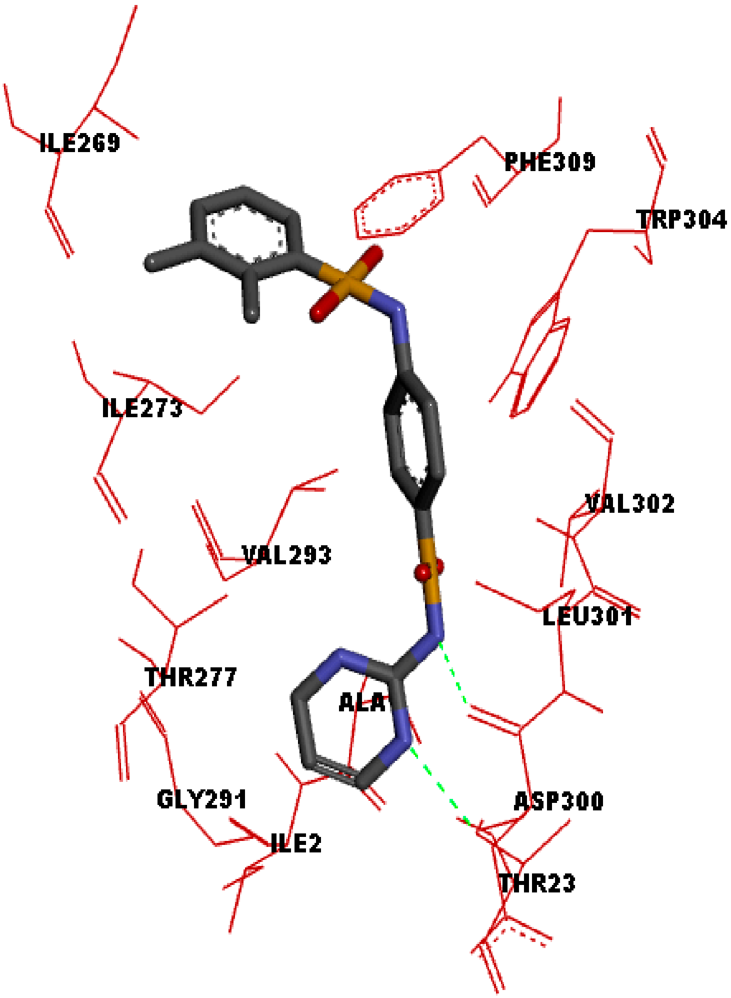

Figure S11. Docking complex **6e**

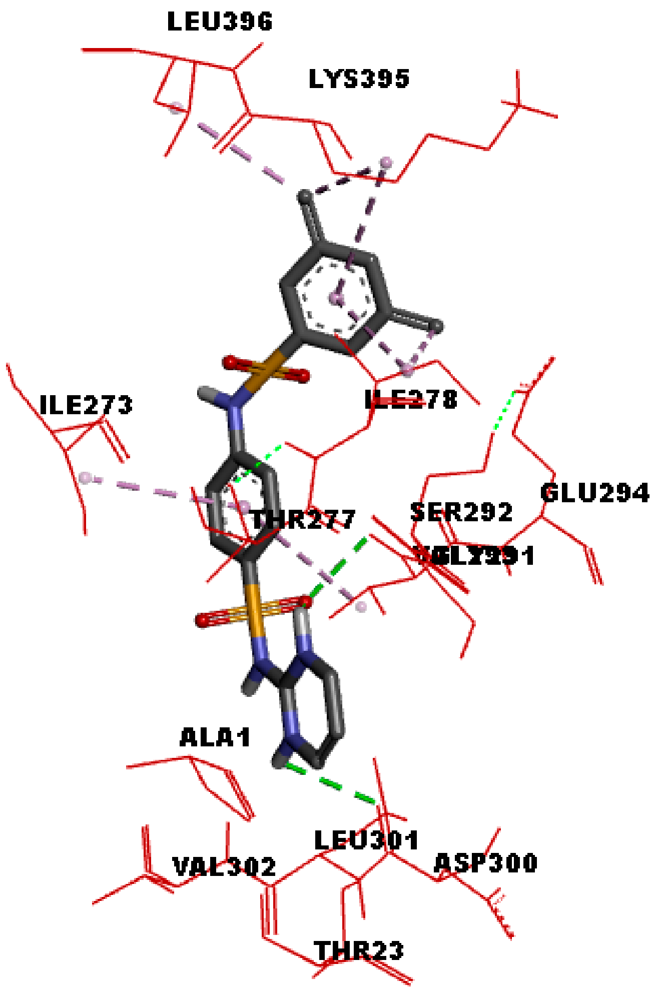

Figure S12. Docking complex **6f**

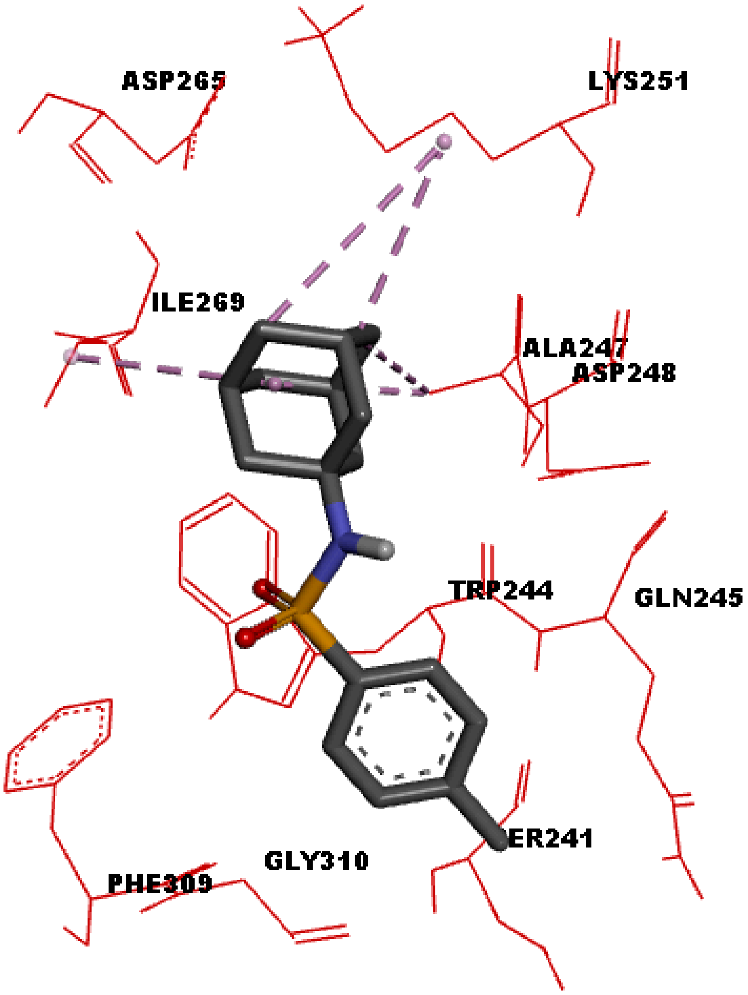

Figure S13. Docking complex **9a**

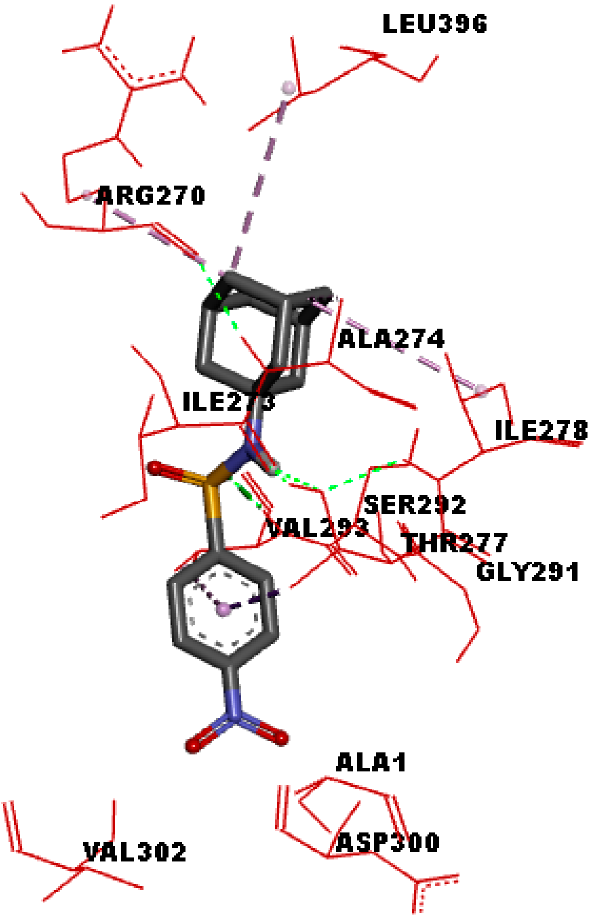

Figure S14. Docking complex **9b**

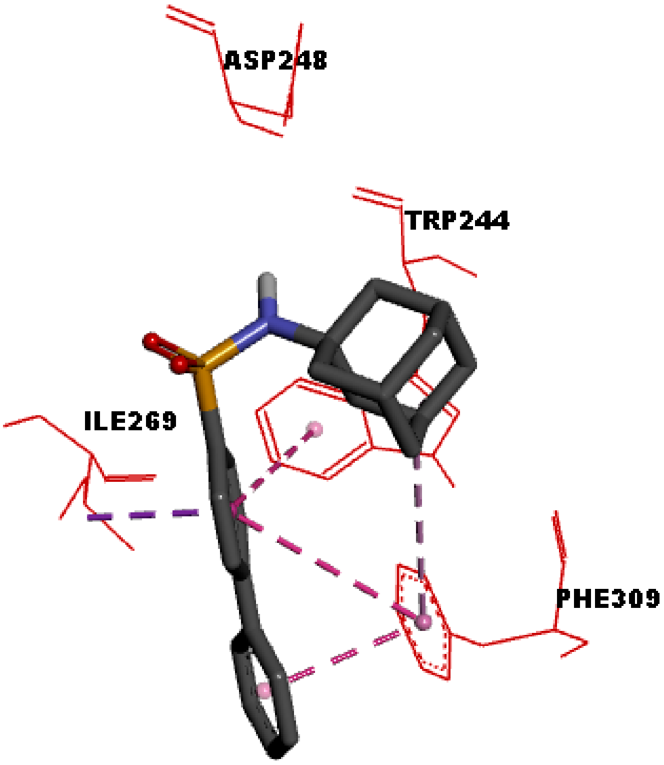

Figure S15. Docking complex **9c**

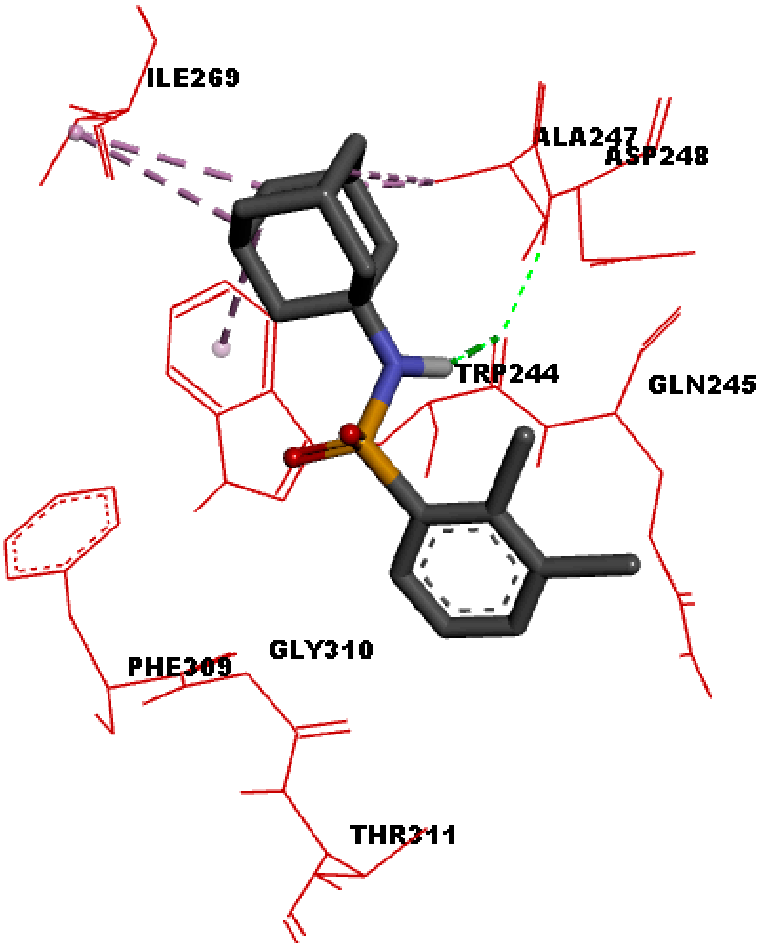

Figure S16. Docking complex **9d**

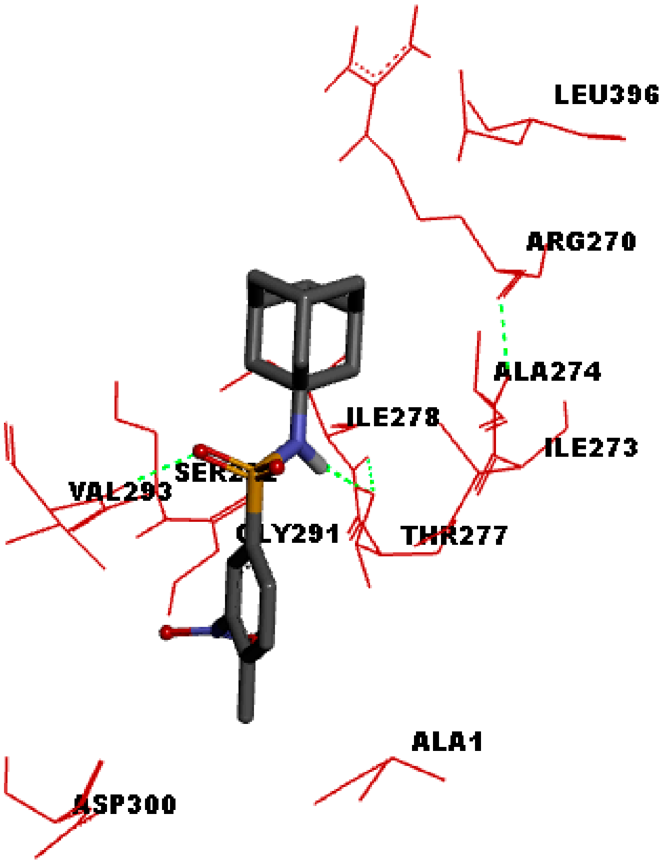

Figure S17. Docking complex **9f**

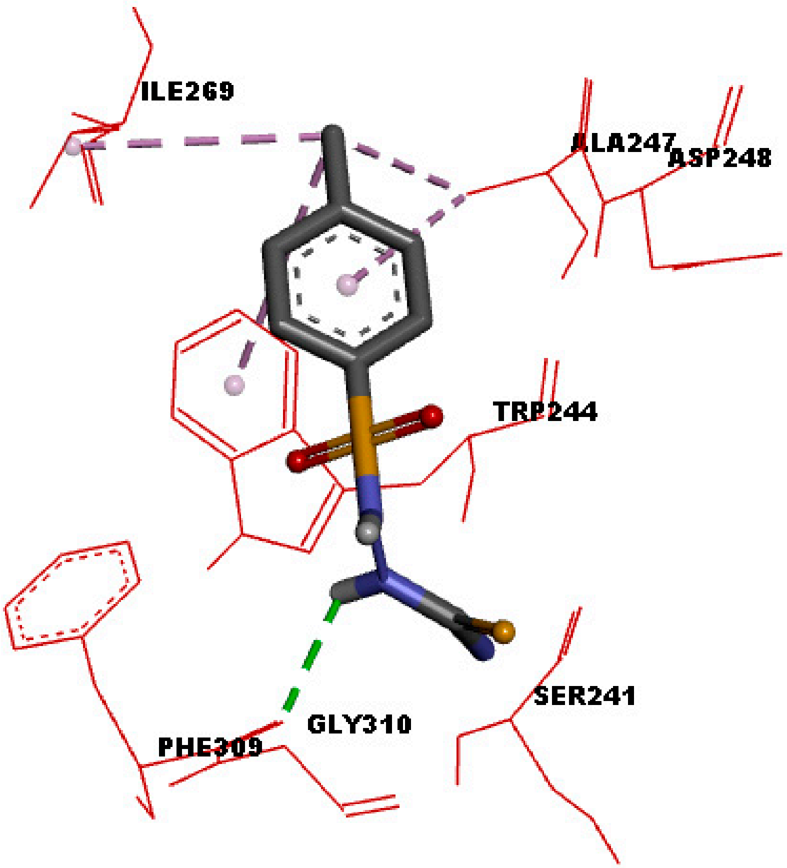

Figure S18. Docking complex **12a**

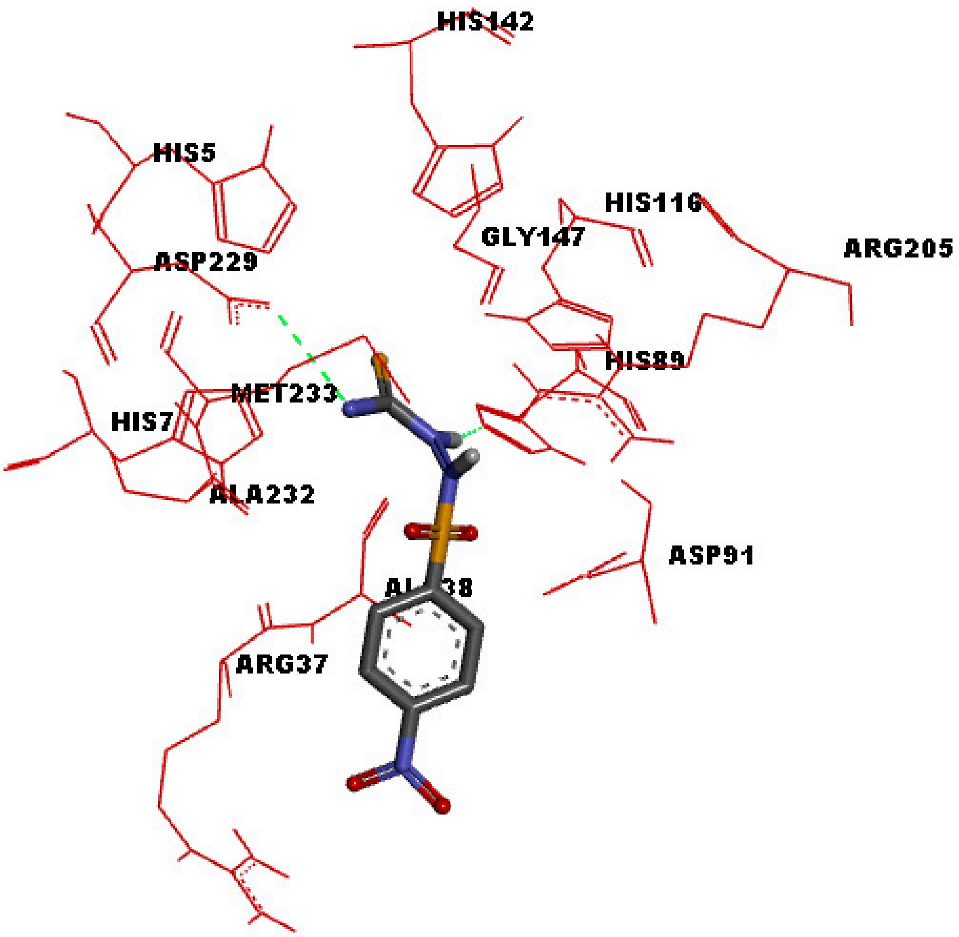

Figure S19. Docking complex **12b**

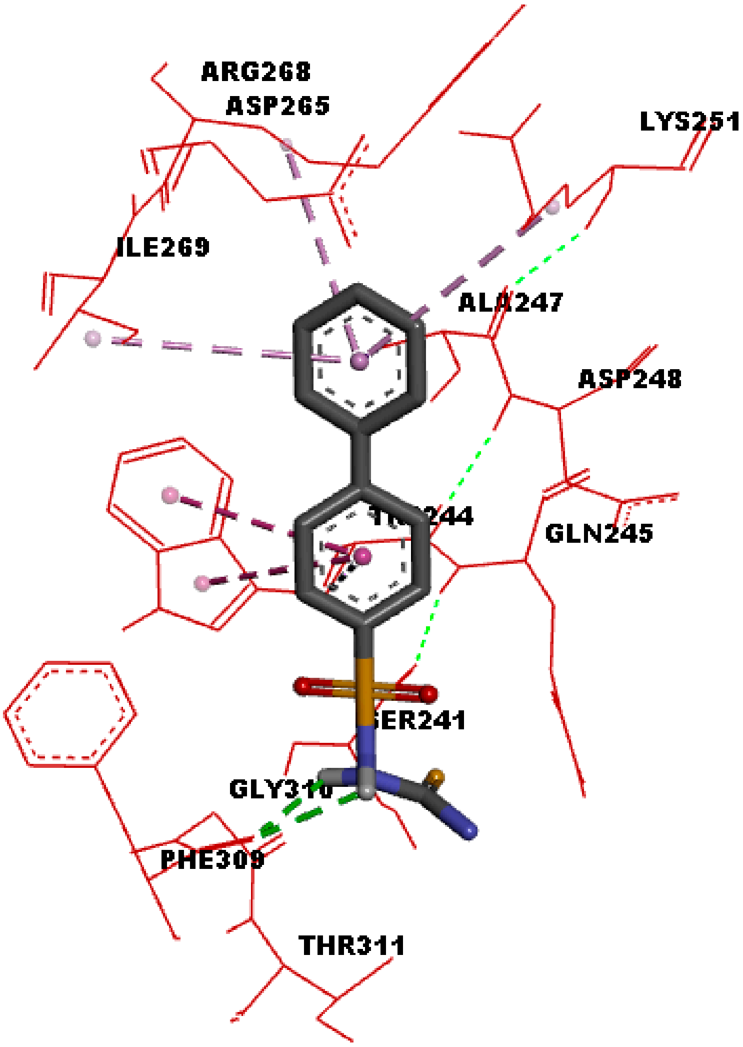

Figure S20. Docking complex **12c**

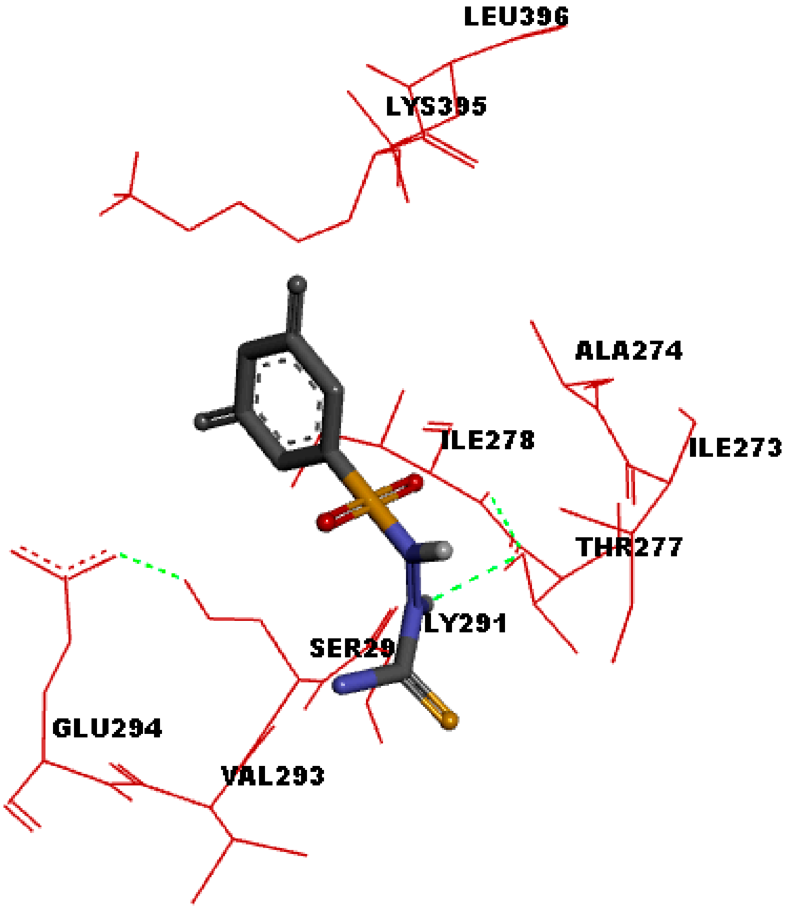

Figure S21. Docking complex **12e**

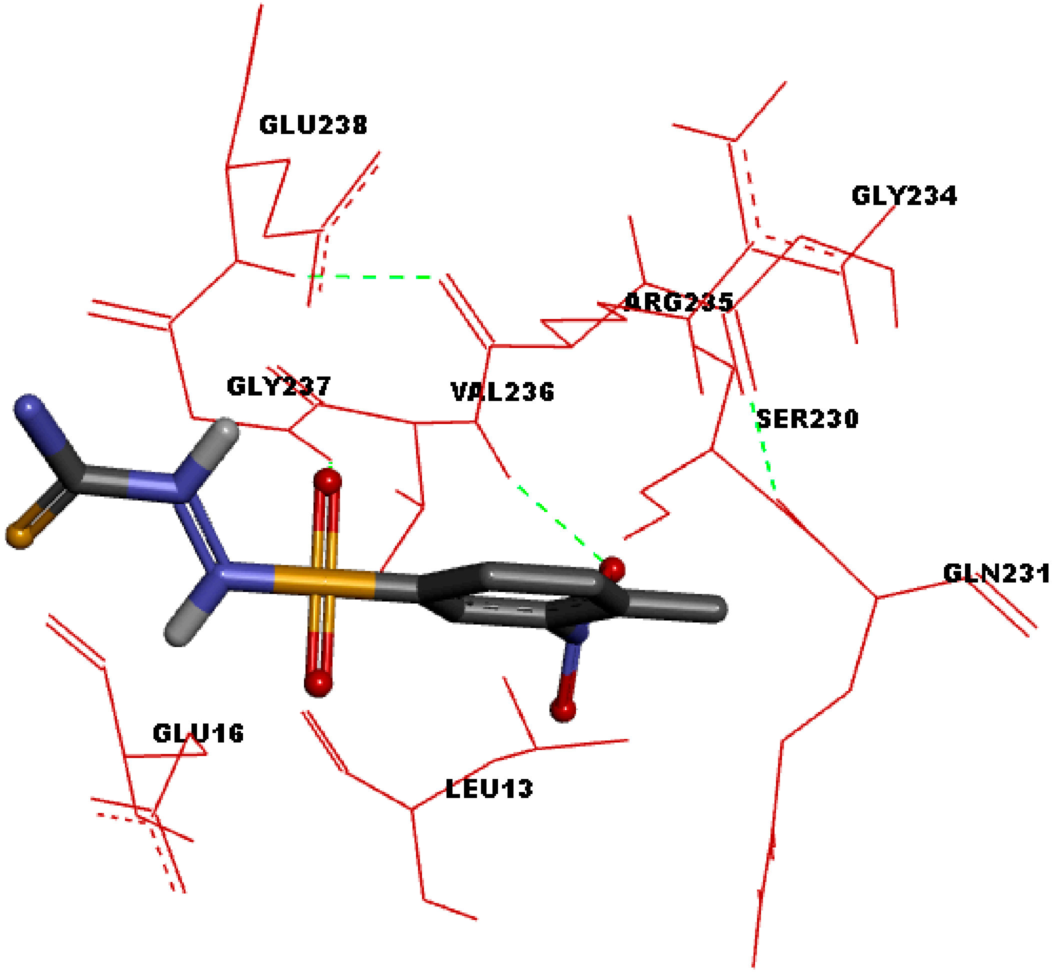

Figure S22. Docking complex **12f**

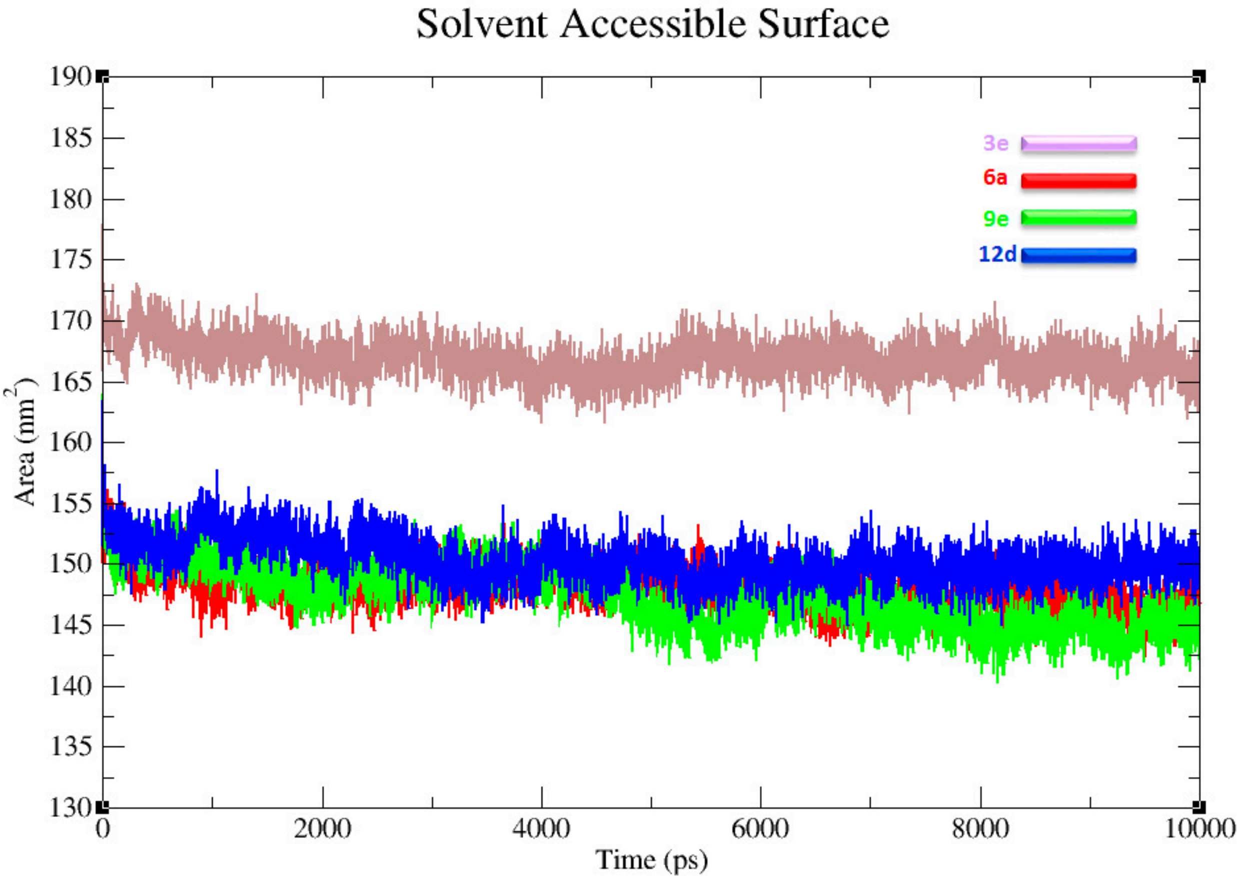

Figure S23: Solvent Accessible Surface Area (SASA) graph of **3e**, **6a**, **9e** and **12d** docked complexes from 0-10000ps time scale.

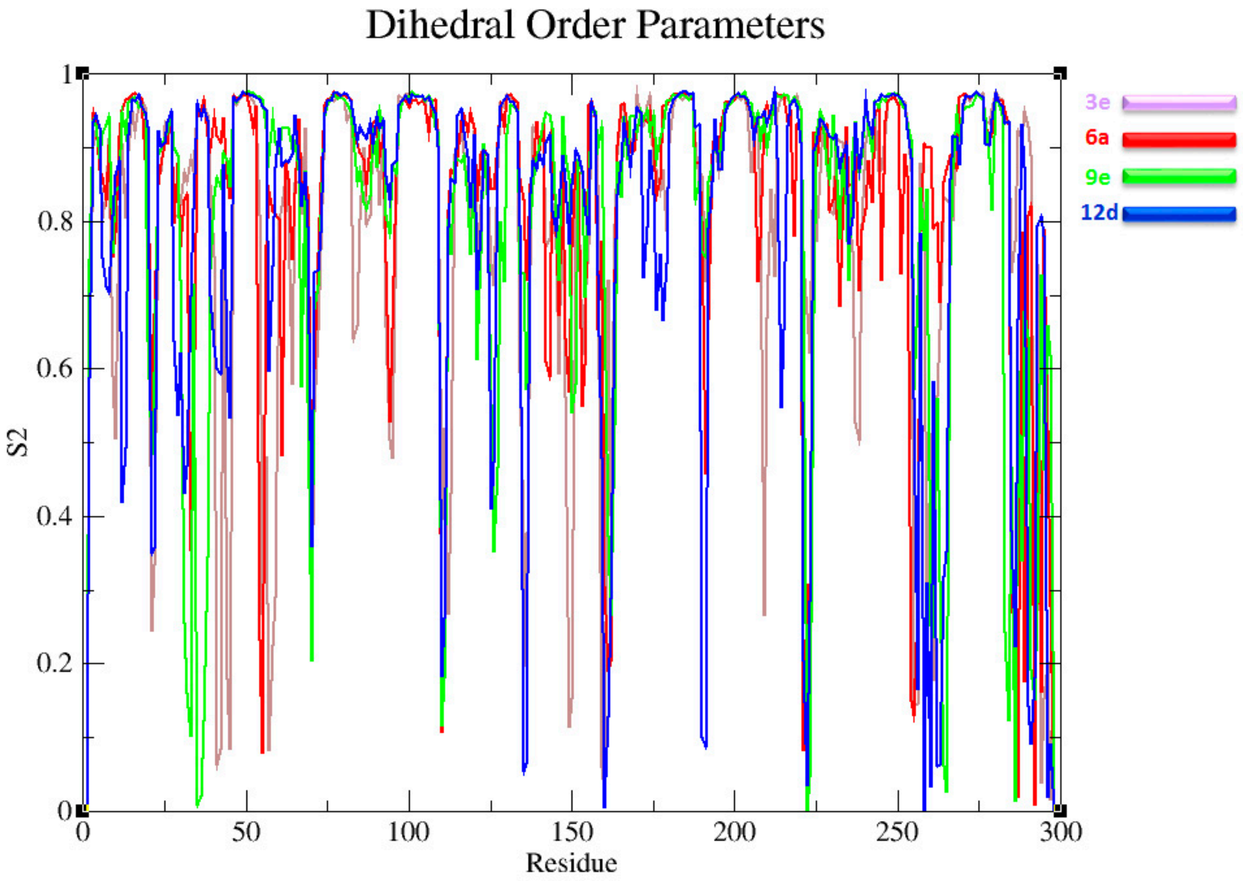

Figure S24. Chi square distribution graph of **3e, 6a, 9e** and **12d** docked complexes from 0-10000ps time scale.

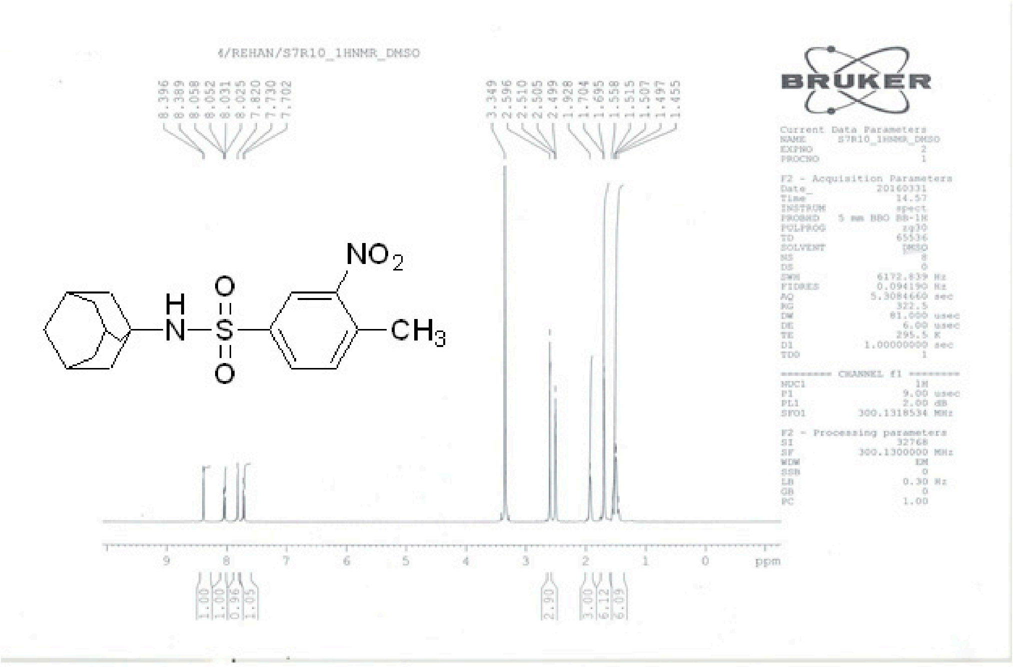

Figure S25. ^{1}H NMR spectrum of **9f**

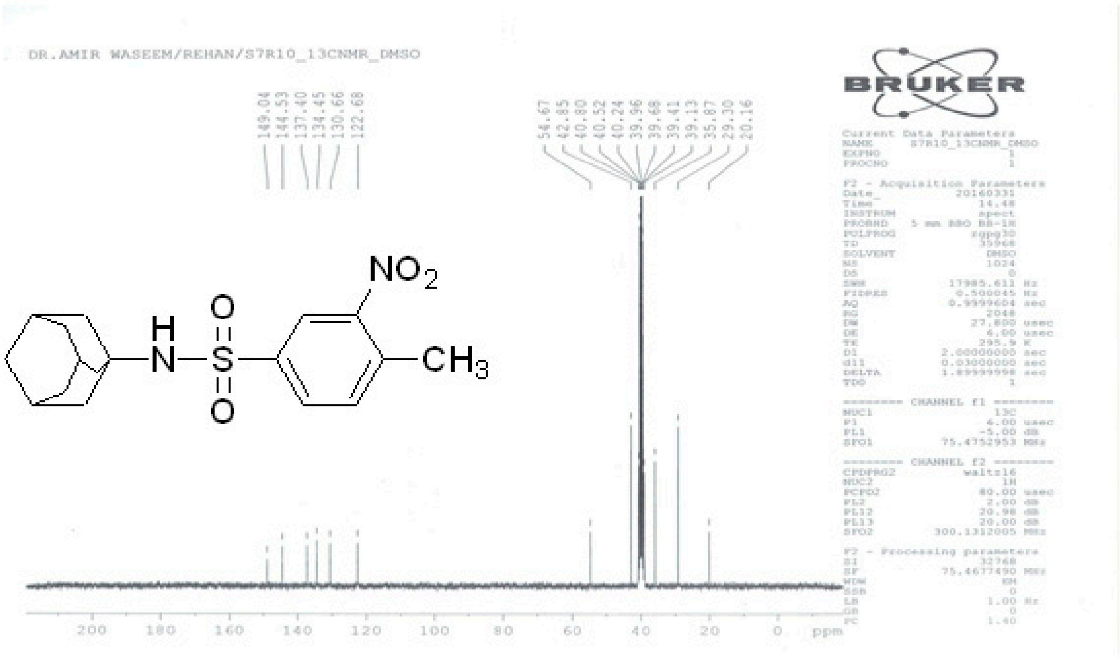

Figure S26. ^{13}C NMR spectrum of **9f**

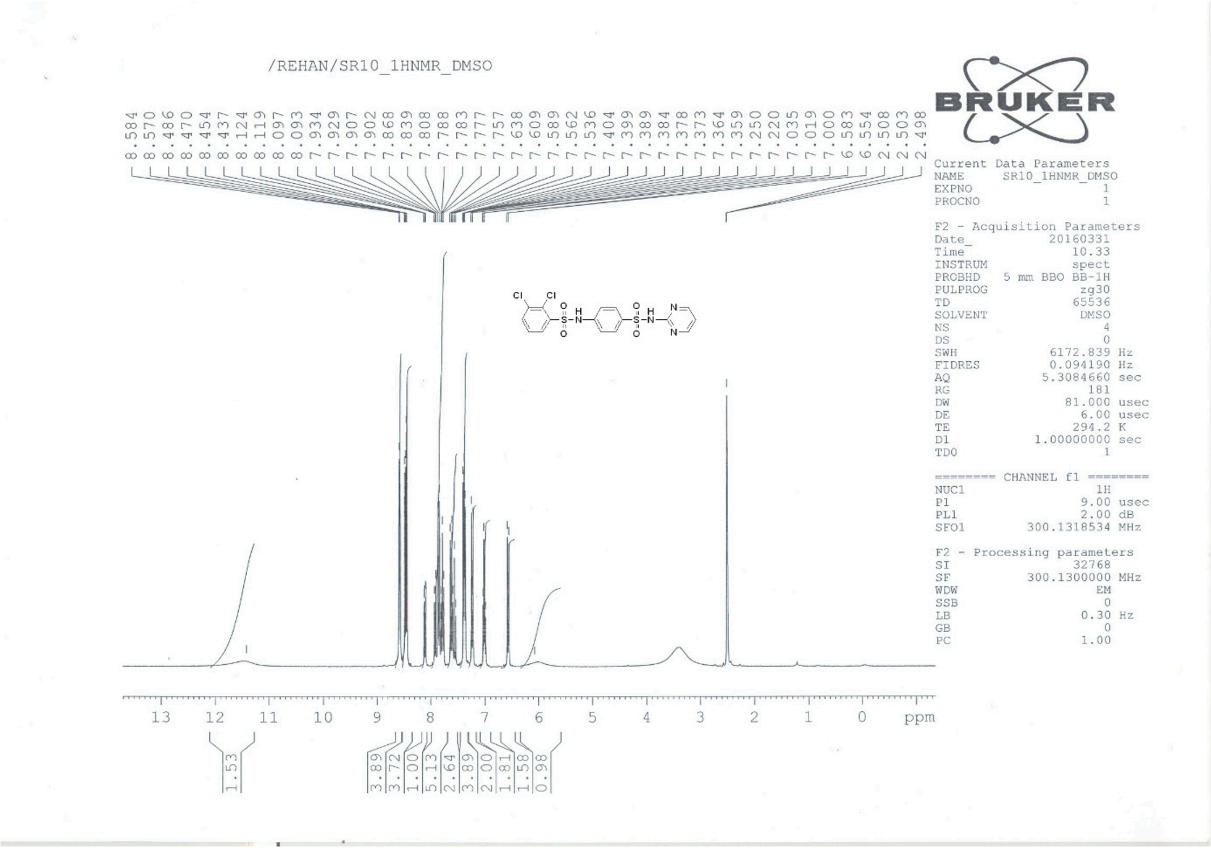

Figure 27. ^{1}H NMR spectrum of **6d**

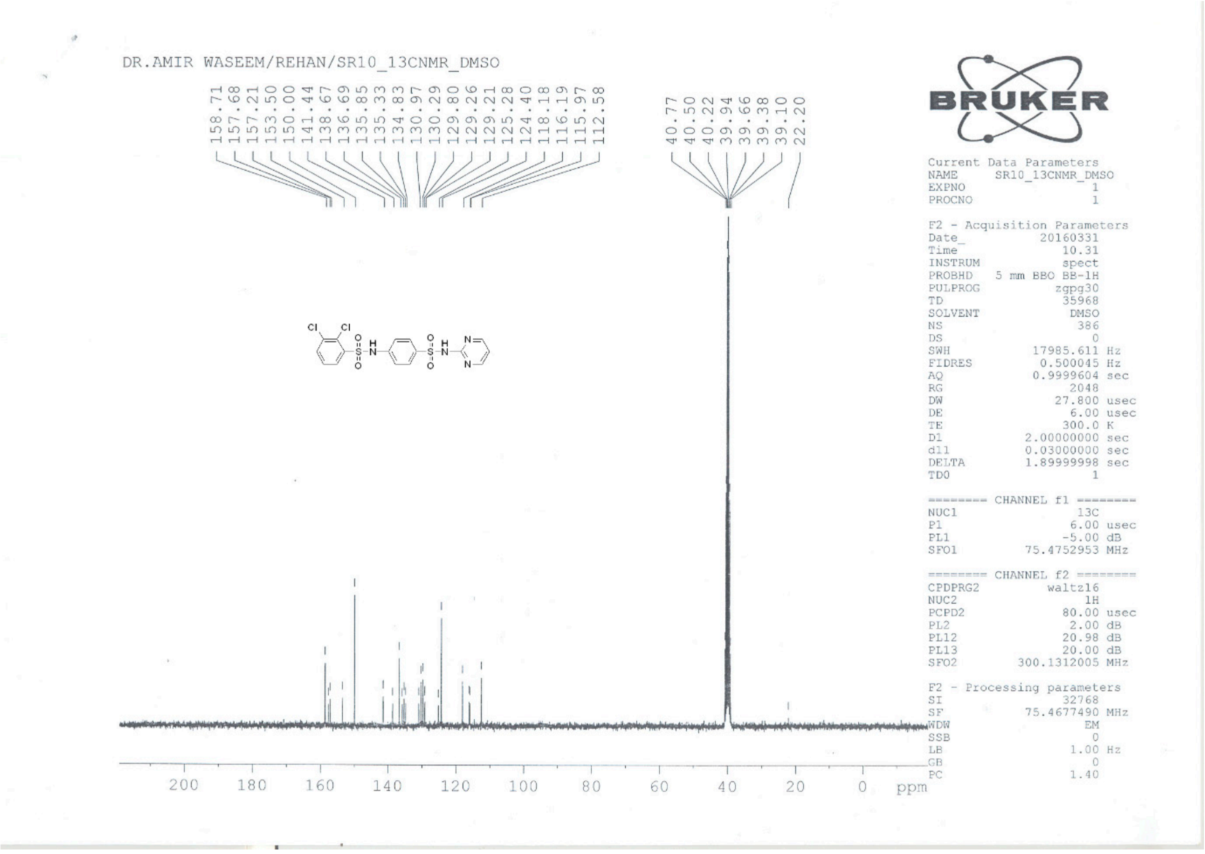

Figure S28. ^{13}C NMR spectrum of **6d**

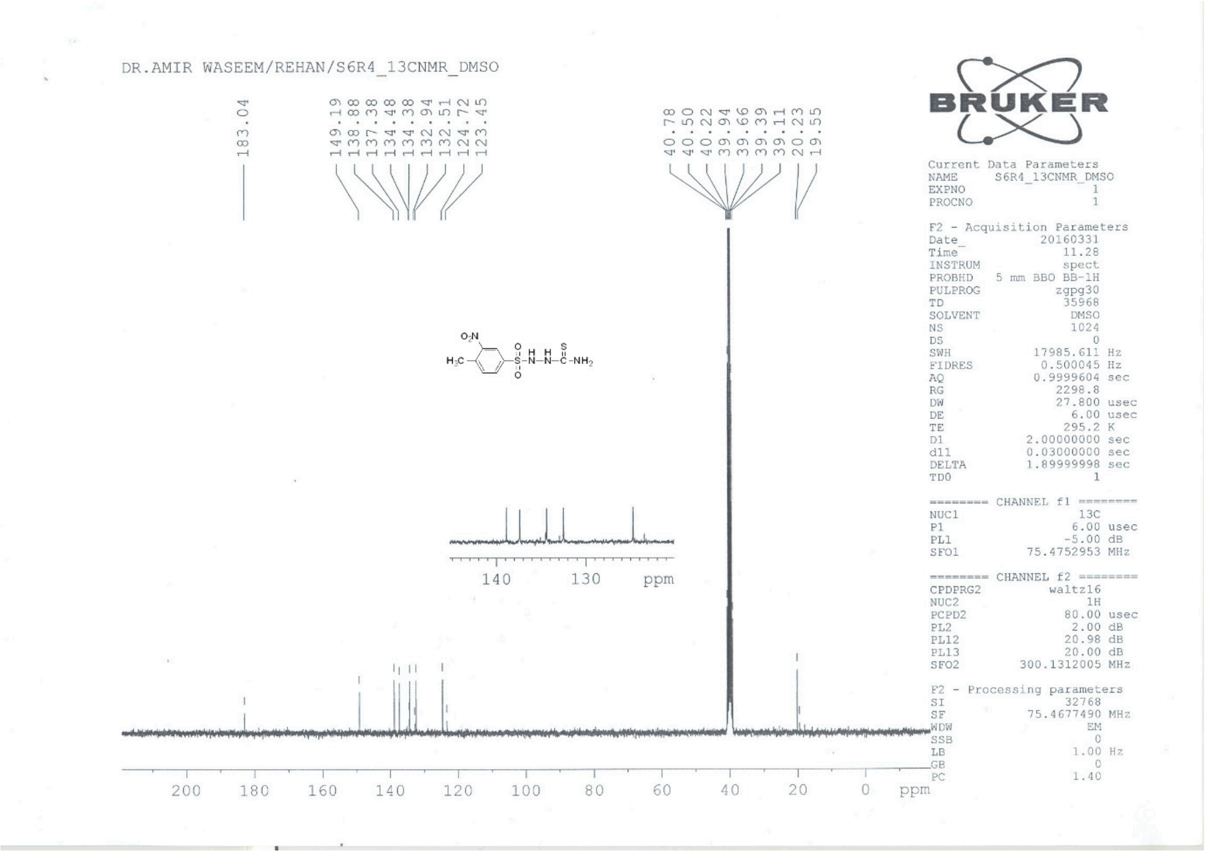

Figure S29. ^{13}C NMR spectrum of **12f**

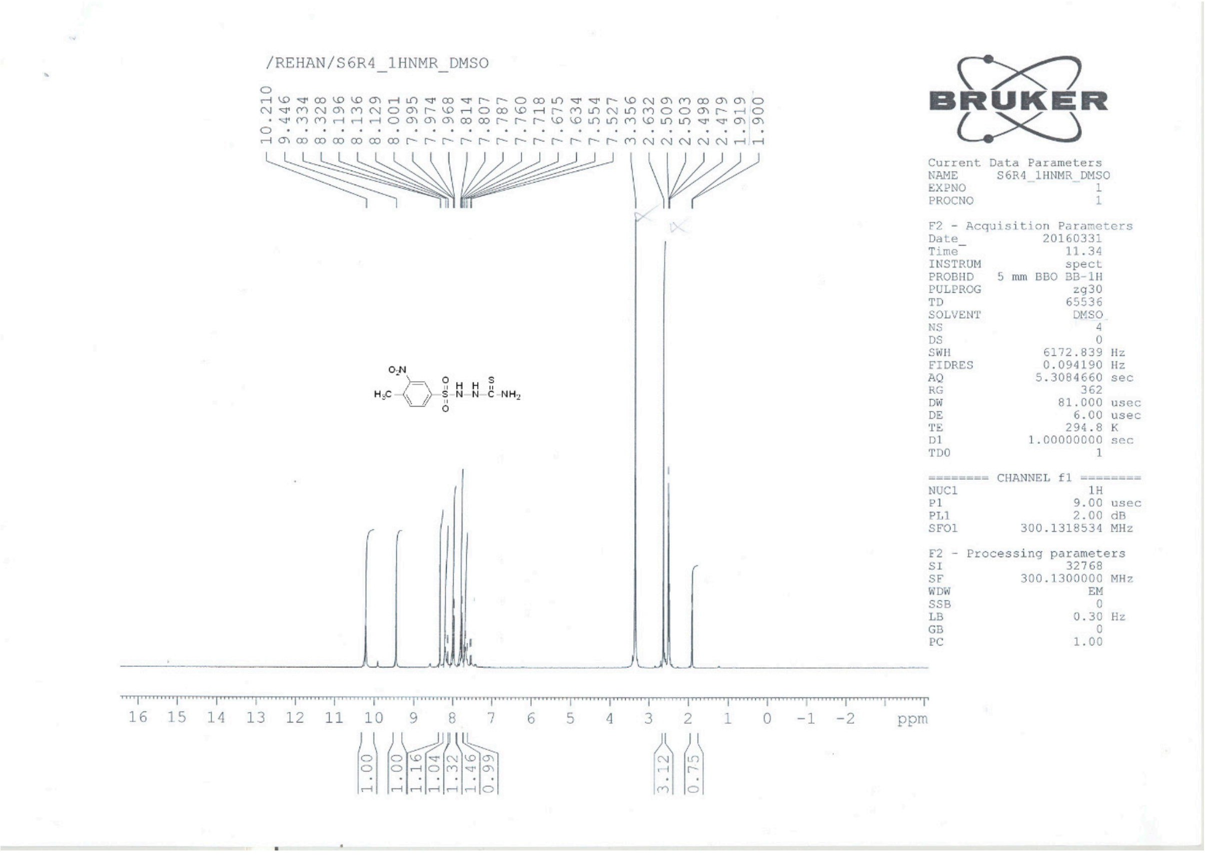

Figure S30. ^{1}H NMR spectrum of **12f**

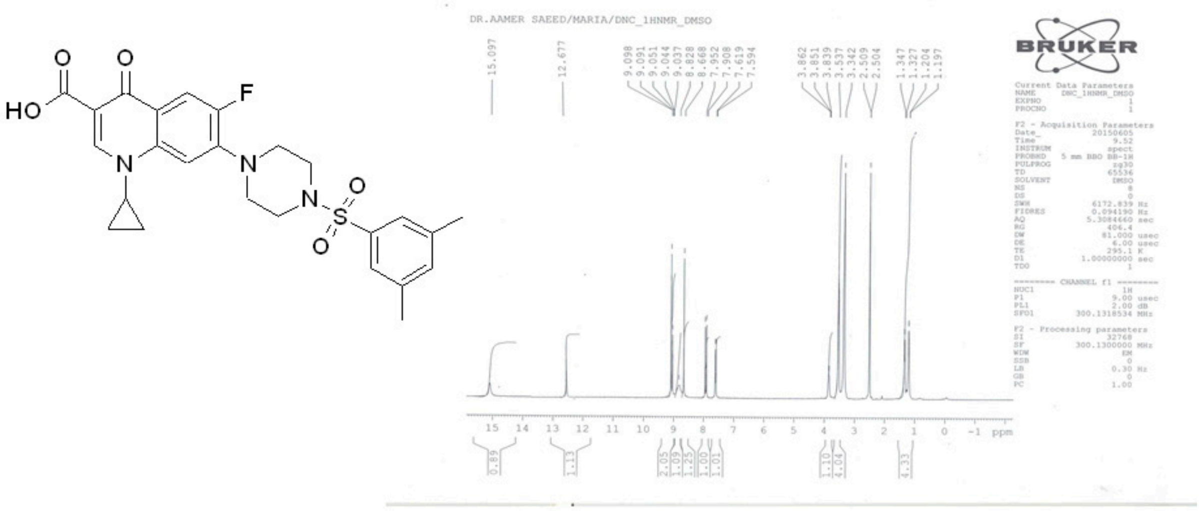

Figure S31. ^{1}H NMR spectrum of **3e**

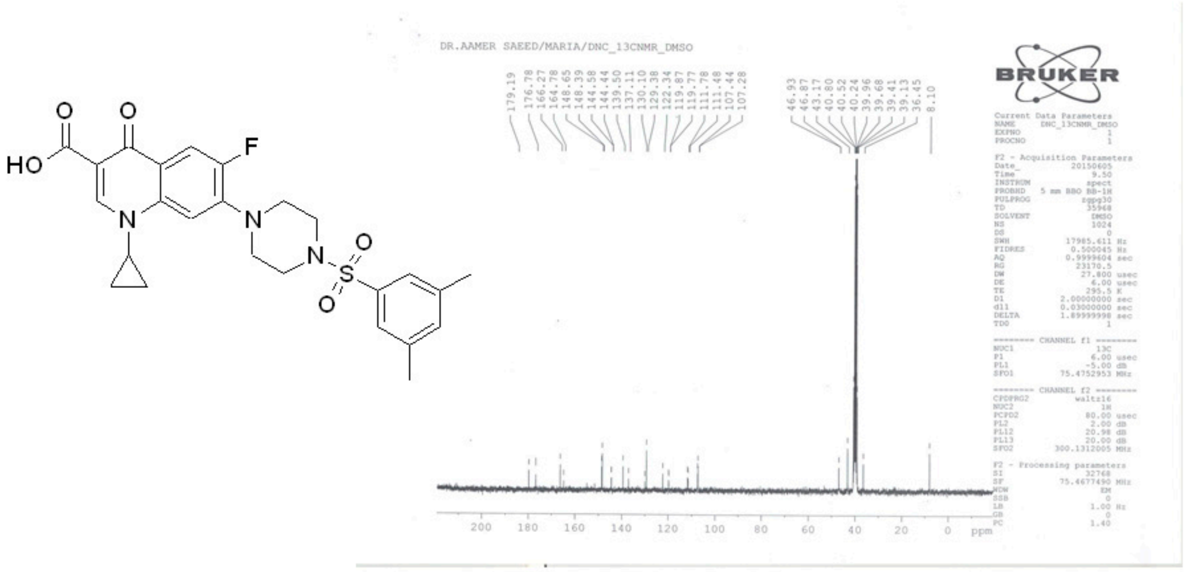

Figure S32. ^{13}C NMR spectrum of **3e**

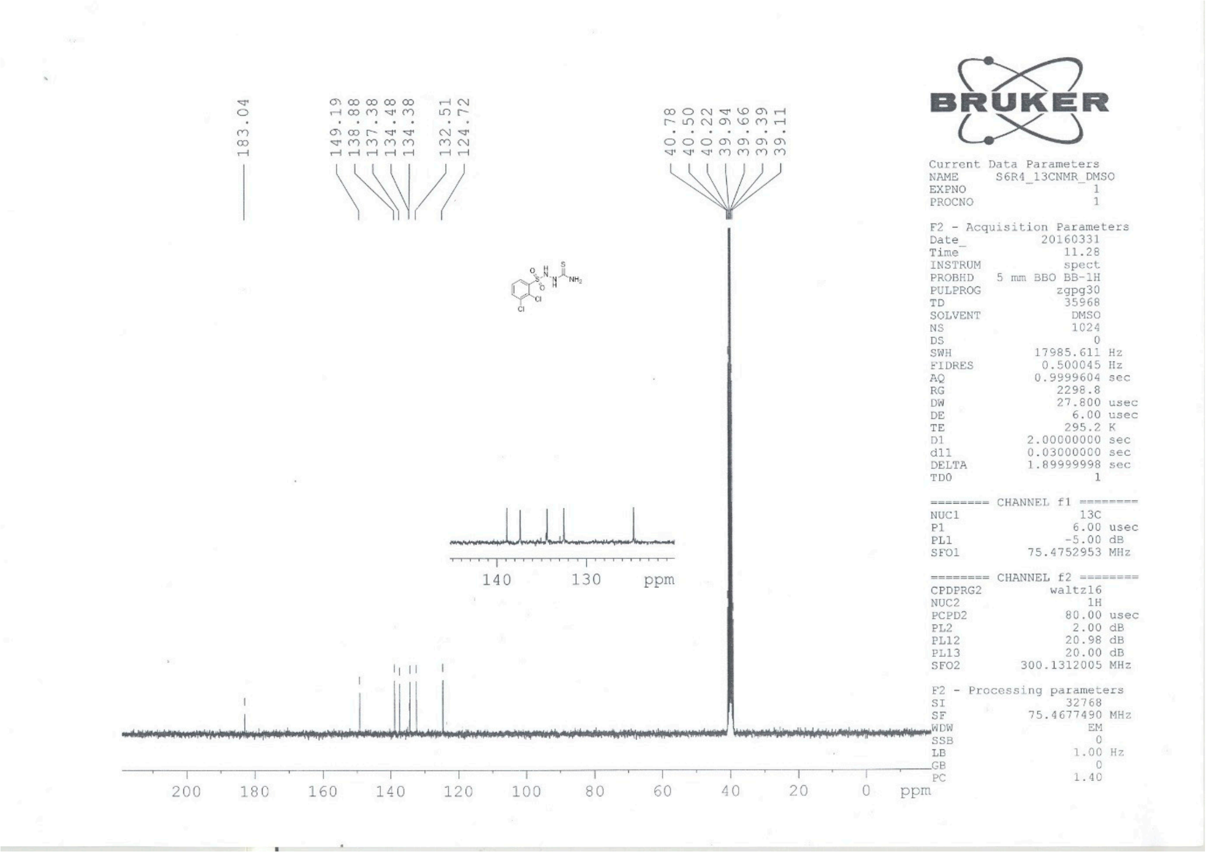

Figure S33. ^{13}C-NMR spectrum of **12d**

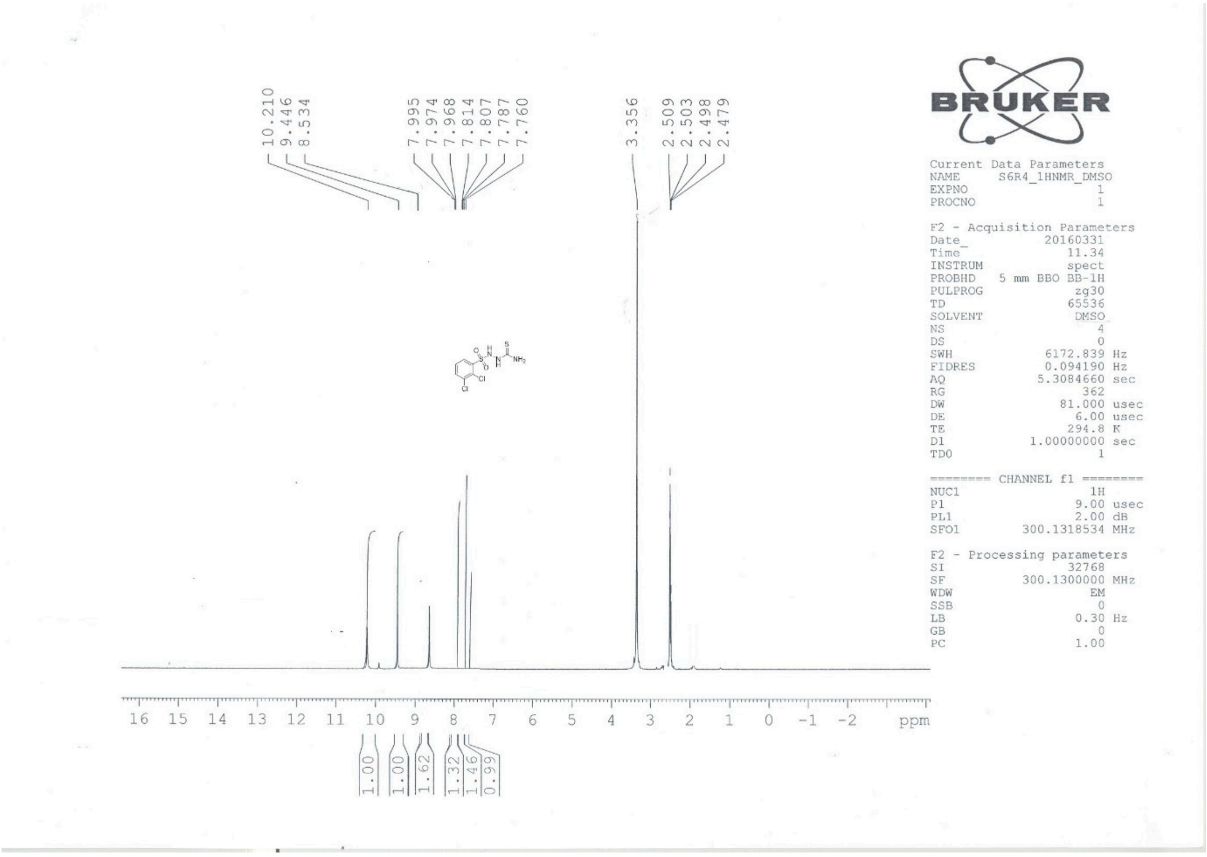

Figure S34. ^{1}H-NMR spectrum of **12d**

3.6 Root mean square deviation and fluctuation (RMSD/RMSF) analysis of targeted protein

Based on docking energy values and in-vitro results, four docked complexes **4e, 6a, 9e** and **12d** were selected to evaluate the residual flexibility of receptor through MD simulation. The RMSD and RMSF graphs were evaluated to determine the protein structural behavior. The RMSD graph result of all docked complexes (**4e, 6a, 9e** and **12d**) interprets the protein residual deviation and fluctuations in 10 ns simulation time frame. Initially, all four graph lines were displayed an increasing trend from 0-2000 ps. However, the RMSD value range from 0.2-0.4 nm for all four complexes. The **3e** and **9e** complexes showed higher fluctuations than the **6a** and **12d** at starting simulation time at 0-2000 ps. After that, from 2000-4000 ps all four complexes graph lines remain stable and showed little fluctuations. At that, both **3e** and **9e** were at a higher level compared to **6a** and **12d** and depicts little bit higher RMSD value. From 4000 to 6000 all four complexes steadily increased with very little fluctuations. However, a big fluctuation difference was observed in all complexes from 6000 to 8000 ps. The **3e** graph line showed decreased fluctuated behavior compared to **9e**. However, both complexes were remained close to each other and depicts no big fluctuation difference. While **6a** and **12d** presented big fluctuation difference at the same time. The 6a showed a less decreasing trend compared to **12d**. After that from 8000 to 1000 ps all four complexes remain stable. The **3e** and **9e** showed more than 0.5 RMSD value while **6a** and **12d** were within that value. The comparative analysis justified that **6a** complex simulation graph is more stable throughout the simulation time period as compared to other complexes. However, their RMSD values has not too much deviant from each other (Fig. 4). The generated RMSF results of all docked complexes (**4e, 6a, 9e** and **12d**) showed

the N to C terminal lobes fluctuations within target protein throughout the simulation period. Initially, the N-terminus loop regions were showing little fluctuations. However, **9e** loop residues showed higher value. The generated graph showed that C-terminal loop region is much fluctuated compared to N-terminus. Result depicted that higher peaks in RMSF graph showed the loop conformation and its fluctuations in the simulation time (Fig. 5).

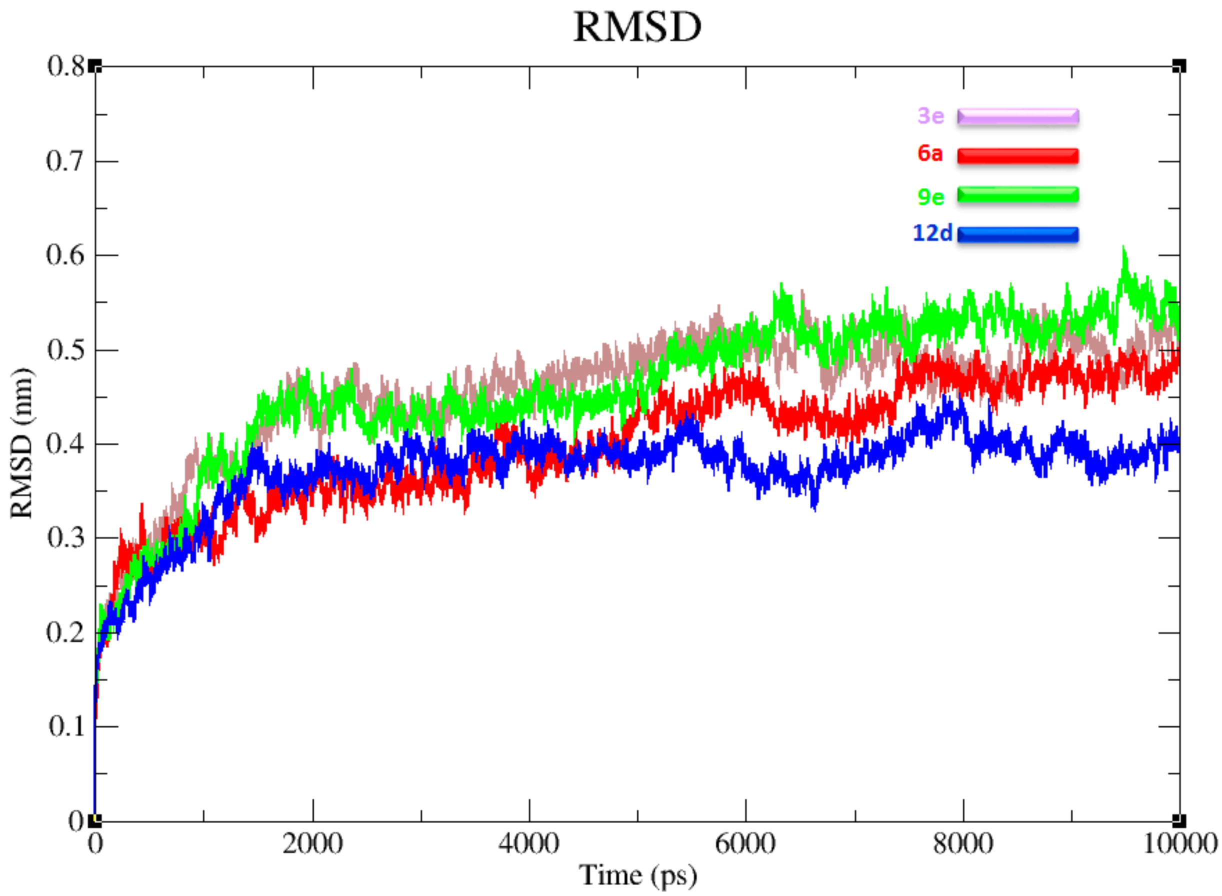

Figure 4. RMSD graph of **3e, 6a, 9e** and **12d** docked complexes are mentioned in purple, red, green and blue colors, respectively from 0-10000ps time scale.

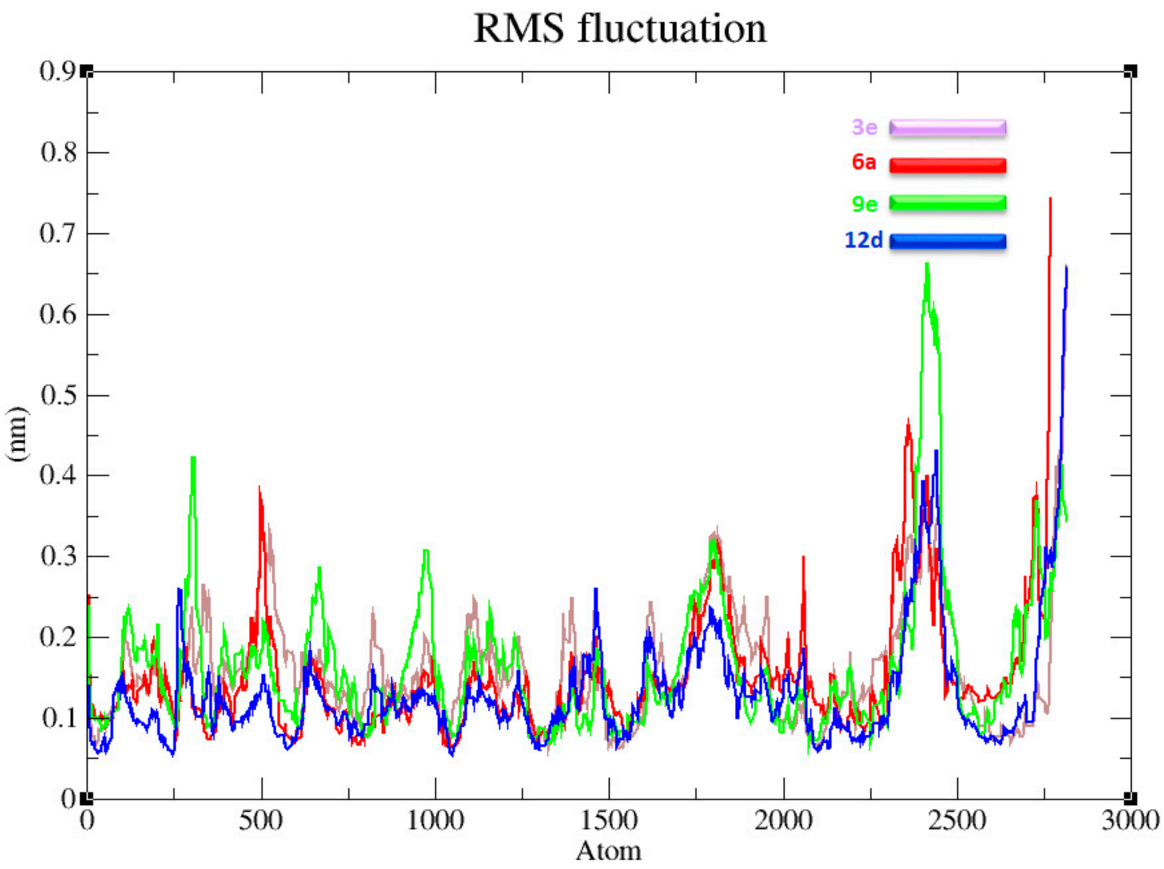

Figure 5. RMSF graph of **3e, 6a, 9e** and **12d** docked complexes are mentioned in purple, red, green and blue colors, respectively from 0-10000ps time scale.
